# Dopey2 and Pcdh7 orchestrate the development of embryonic neural stem cells/ progenitors in zebrafish

**DOI:** 10.1016/j.isci.2023.106273

**Published:** 2023-02-25

**Authors:** Yue Xiao, Min Hu, Qiyan Lin, Ting Zhang, Siying Li, Linjuan Shu, Xiuli Song, Xiaoyong Xu, Wentong Meng, Xue Li, Hong Xu, Xianming Mo

**Affiliations:** 1 Department of Pediatric Surgery, Laboratory of Stem Cell Biology, State Key Laboratory of Biotherapy, West China Hospital, West China Medical School, Sichuan University, Chengdu 610041, China; 2 Hangzhou HuaAn Biotechnology Co.Ltd, Hangzhou, China

**Keywords:** Biological sciences, Neuroscience, Molecular neuroscience, Developmental neuroscience

## Abstract

DOPEY2 has been shown to be associated with Down syndrome and PCDH7 might be involved in Rett syndrome and *MECP2* duplication syndrome. The mechanism how both proteins play roles in these syndromes are largely unknown. Here, we show that Dopey2 and Pcdh7 balance the proliferation and differentiation of neural stem cells and progenitors during embryonic neurogenesis to generate proper size and architecture of zebrafish brains. Dopey2 and Pcdh7 mutually restricted expression of each other in zebrafish embryos. Dopey2 was responsible for the proliferation of neural stem cells/progenitors, whereas Pcdh7 was responsible for the differentiation of neural stem cells/progenitors. Both proteins were shown to orchestrate the proper development and arrangement of neural cells in zebrafish embryonic brains. The results provide an insight into mechanisms to understand how the embryonic brain is constituted and how developmental defects occur in the brains of patients with Down syndrome, Rett syndrome, or *MECP2* duplication syndrome.

## Highlights

• Dopey2 controls zebrafish embryonic the proliferation of neural stem/progenitor cells
• Pcdh7 promotes neural differentiation during zebrafish development
• Dopey2 and Pcdh7 mutually restricted the expression of each other
• Dopey2 and Pcdh7 orchestrate the proper neural development in zebrafish embryos

## Graphical abstract

**Figure:**
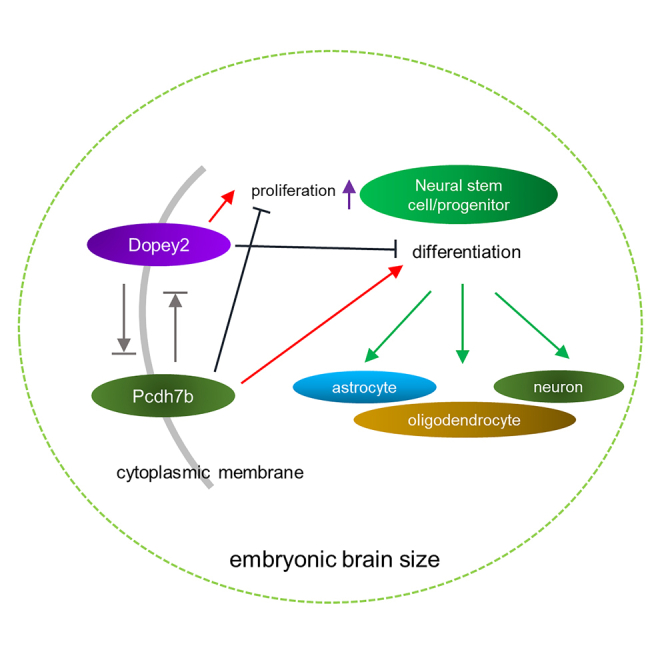

## Introduction

*DOPEY2* is localized within the chromatin region that is critical for Down Syndrome (DS) and has been considered to be a candidate gene responsible for neuromorphological alterations and mental retardation in DS.^1^ Neuromorphological features in DS patients include hypoplasia of the hippocampus, cerebral cortex, larger parahippocampal gyrus, and smaller cerebellum. Infants with DS have been shown to possess abnormal cortex development and delayed brain maturation. They also carry fewer neurons and have a lower neuronal density in their brains.^2^^,^^3^ An *in situ* quantitative assessment of gene expression has shown that *DOPEY2* is overexpressed more than 50% in the cerebral cortex, cerebellum, and the hippocampus, respectively. This finding is in agreement to the hypothesis that *DOPEY2* plays a potential role in functional brain alterations and in the pathogenesis of mental retardation in Down syndrome.^4^ However, how DOPEY2 modulates the neural development and brain maturation remains unknown.

Rett syndrome (RTT) is an early onset neurodevelopmental disorder with severe cognitive and physical disabilities caused by mutation in the X-linked gene methyl-CpG-binding protein 2 (*MECP2*),^5^ a ubiquitously expressed transcriptional regulator. Duplication of the *MECP2* locus causes *MECP2* duplication syndrome (MDS), a severe neurodevelopmental disorder.^6^^,^^7^ Despite remarkable scientific progress because the discovery of both disorders, the mechanisms of how MECP2 dysfunctions lead to both RTT and MDS is largely unknown. Protocadherin (PCDH) 7 belongs to δ-protocadherin family and is predominantly expressed in the brain and heart and highly expressed in neurons and astrocytes.^8^ The mRNA level of *PCDH7* is down regulated by MeCP2^9^^,^^10^ and up regulated by the reduction of MeCP2 functions. These observations suggest that PCDH7 might be involved in Rett syndrome and *MECP2* duplication syndrome.

Both DOPEY2 and PCDH7 are membrane proteins able to sense and mediate extrinsic cues in cells. Extrinsic cues regularize cell fate specification and differentiation decisions during the embryonic development of brains.^11^ The most important extrinsic cues are morphogenic signals which pattern the neuroepithelium into discrete progenitor domains and modulate the proliferation and differentiation of neural stem cells and progenitors to constitute brain structures.^12^^,^^13^^,^^14^^,^^15^ Other components of the extrinsic environment of neural stem cells and progenitors, including the surrounding cell types, cell-to-cell interactions, the extracellular matrix, the basal lamina, are also referred to be involved in embryonic and adult neurogenesis in brains.^16^^,^^17^ Despite this inherent complexity of extrinsic cues, accurate incorporation of signaling must take place within the neural stem cells and progenitors for appropriate development of the embryonic brains. Cellular membrane proteins are the first places to sense extrinsic cues and initiate the process of extrinsic signal integration.^18^ Although roles for extrinsic signals in embryonic brain development are well established, our understanding of the members and roles of the membrane proteins in neural stem cells and progenitors during the embryonic neurogenesis is limited. In the present work, we identify two membrane proteins that balance the proliferation and differentiation of the neural stem cells and progenitors for the proper size and neural cell arrangement of brain during zebrafish embryonic development.

## Results

### Dopey 2 is expressed in neural stem cells/progenitors of mouse and zebrafish embryos

We screened more than 70 monoclonal antibodies generated by the injection of mouse embryonic stem cells (ESC) into mice^19^ and identified one antibody against Dopey2 protein on the surface of mouse ESC (data not shown). Immunofluorescence staining showed that Dopey2 antibody stained the cells within the ventricular zone (VZ) and the sub-ventricular zone (SVZ) marked by the expression of Sox2, a marker for neural stem cells and progenitor cells in mouse embryonic brains (Figure S1A). In addition, the antibody nicely stained cells in zebrafish embryos, as seen in Figure S1B. The staining showed that Dopey2 protein was found on the surface of zebrafish embryonic cells from one-cell-stage of embryos (Figure S1B). Further detection showed that Dopey2 protein is mainly located in the regions of zebrafish embryonic brains, consistent with the results derived from the staining of mouse embryonic brains (Figures S1A and 1B). The results also indicate that mouse Dopey2 is the conserved homologue of zebrafish Dopey2 protein. The temporal and spatial expressing patterns of *dopey2* gene is both maternally and zygotically expressed and has high expressing levels in zebrafish embryonic brains (Figures S1C–S1E).

**Figure 1:**
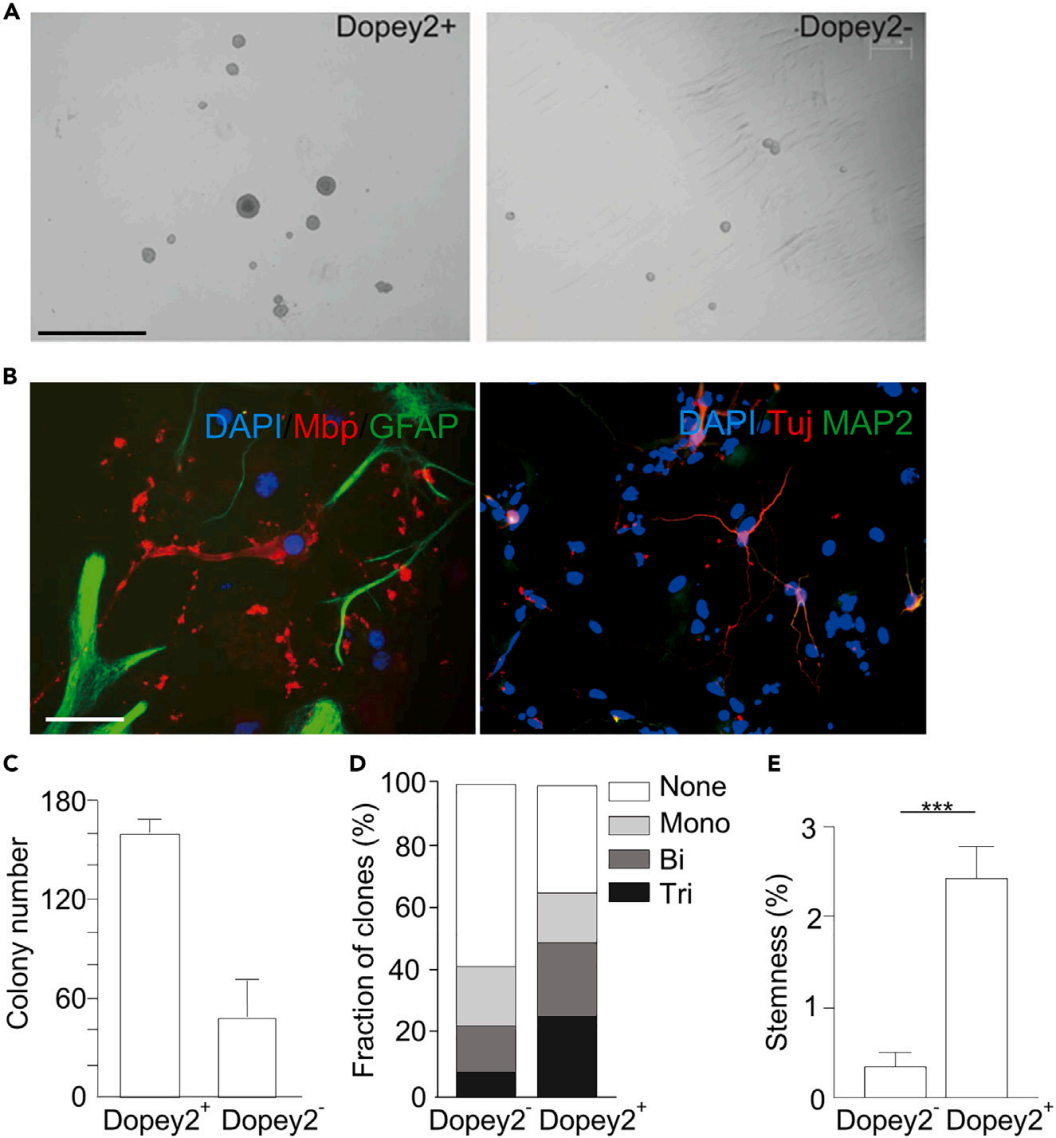
Dopey2+ cells exhibit higher stemness capacity (A) Fluorescence-activated cell sorted Dopey2^+^ and Dopey2^-^ NSCs/neural progenitor cells were seed in semi-solid NSC medium. After 7 days, Dopey2^+^ NSCs/neural progenitor cells formed larger clones. Scale bar = 1 mm. (B) In NCFC assay, neurospheres cultured as monolayer for differentiation were multi-labeled immunostained for DAPI/Mbp/GFAP and DAPI/Tuj/MAP2. Scale bar = 50 μm. (C) Colony number of Dopey2^+^ and Dopey2^-^ neuroshperes (means ± SEM; n = 3). (D) Neurospheres were measured and grouped. Tripotential clone: differentiating into three types of neural cells; bipotential clone: differentiating into either two cell types; monopotential clone: differentiating into one cell type. (E) Stemness calculated by dividing the number of forming tripotential clones by total cells. Student’s *t* test: ∗∗∗p < 0.001 indicates a significant increase when Dopey2^+^ group compared with Dopey2^-^ group.

To evaluate the function of Dopey2 in neural cells, we used fluorescence-activated cell sorting approach to isolate Dopey2^+^ neural cells from the brain of E15 mouse embryos and cultured them in semi-solid medium. The Dopey2^+^ neural cells formed more colonies under neural culture conditions (Figure 1). Then we performed a neural colony-forming cell assay, which is an established approach to discriminate neural stem cells from more committed neural progenitors.^20^^,^^21^^,^^22^ Under conditions promoting proliferation, more than 20% of seeded Dopey2^+^ cells formed tripotential clones (Figures 1B and 1D). Less than 10% of the Dopey2^-^ cells formed tripotential clones. We assessed the frequency of stemness by determining the self-renewal and tripotential capacity of the secondary neurospheres. The number of neural stem cells was 5-fold higher in the Dopey2^+^ cell population (Figure 1E). The results indicate that Dopey2 expressed neural cells contain neural stem cells in mouse embryonic brains.

### Dopey2 is involved in the brain development in zebrafish embryos

**Figure 2:**
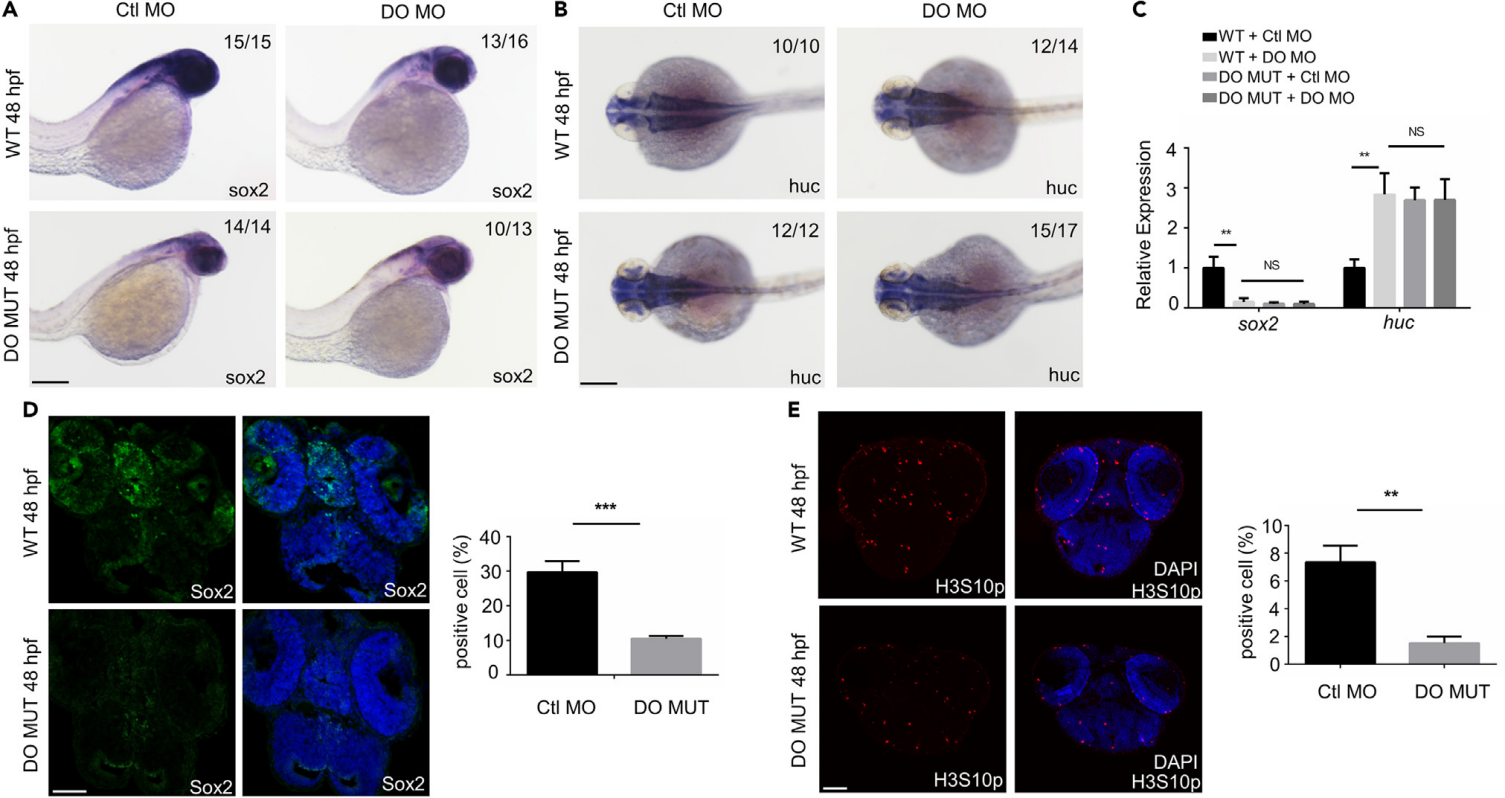
Dopey2 modulates the proliferation of neural stem cells/progenitors and inhibits the differentiation of neural stem cells/progenitors in zebrafish embryonic brains (A and B) *In situ* hybridization analysis showed the expression of *sox2*, *huc* genes. Embryos were analyzed at 48 hpf (hour postfertilization). WT or DO MO embryos were pre-injected with Ctl MO or DO MO. Scale bar = 200 μm. (C) Real-time fluorescence quantitative PCR analysis showed the relative expression level of *sox2*, *huc* genes in embryos at 48 hpf, WT or DO MO embryos were pre-injected with Ctl MO or DO MO (mean ± s.e.m, n = 3, Student’s *t* test: ∗∗∗p < 0.001, ∗∗p < 0.01, ∗p < 0.05, NS = not significant). (D) Representative immunofluorescence staining for Sox2 in frozen sections of 48 hpf WT or DO MUT embryos. Scale bar = 50 μm. Diagram showed quantitative analysis of Sox2 positive cells in WT or DO MUT embryos (mean ± s.e.m, n = 3, Student’s *t* test: ∗∗∗p < 0.001). Images were quantified by ImageJ software. (E) Representative immunofluorescence staining of H3S10p in the frozen sections of 48 hpf WT or DO MUT embryos. Scale bar = 50 μm. Diagram showed quantitative analysis of H3S10p positive cells in WT or DO MUT embryos (mean ± s.e.m, n = 3, Student’s *t* test: ∗∗p < 0.01). Images were quantified by ImageJ software. Ctl MO: control MO, DO MO: *dopey2* MO, WT: wild type, DO MUT: *dopey2*mutant.

To identify the function of Dopey2 during the embryonic development, an antisense morpholino oligonucleotide (DO MO) complementary to the translational initiation site of zebrafish *dopey2* mRNA (Figure S2A) and N-terminal and C-terminal deleted mRNA mutants of *dopey2* were injected into zebrafish embryos (Figure S2B). The results showed that DO MO effectively blocked the expression of Dopey2 protein (Figures S2A, S2D, andS2E) and induced defective phenotypes in the brains of zebrafish embryos S3A, B, C). The defective brain phenotypes of the morphants were restored by co-injection of capped misRNA (mRNA with mismatched bases) of the zebrafish *dopey2* with synonymous mutations at the MO binding sites (Figure S3C). The embryos injected with truncated *dopey2* mutants displayed the highly similar brain phenotypes in zebrafish embryos (Figure S3C). The results also demonstrate that the Dopey2 mutants with N-terminal or C-terminal deletion display the dominant-negative interfering paralogous (DNIP) of Dopey2 protein and can disrupt the function of wild type Dopey2 protein *in vivo*. Finally, we generated *dopey2* gene mutant zebrafish (DO MUT) using CRISPR/Cas9 system (Figures 2A–2C, and S2C). In embryos injected with DO MO or DO MUT embryos, the level of Dopey2 protein is significantly decreased or diminished (Figure S2E). DO MUT embryos displayed defective brain phenotypes that highly liked ones caused by injections of DO MO and truncated *dopey2* RNAs (Figure S3D). Injection of DO MO into DO MUT embryos did not display any additional defects, confirming the efficiency and credibility of DO MO (Figures 2A–2C). The results demonstrate that Dopey2 is involved in the brain development in zebrafish embryos.

### Dopey2 is responsible for the proliferation of embryonic neural stem cells/progenitors

Injection of the control MO (Ctl MO), DO MO or truncated *dopey2* RNA into zebrafish embryos at the 1-cell stage was performed. The injected embryos presented with narrower or closed ventricles of midbrains and hindbrains (Figures S3A andS3B). We confirmed the phenotypes of these zebrafish embryonic brains using expressing patterns of marker genes, including *zic1*, *fgf8a*, *pax2a*, *gfap*, *nestin*, *sox2*, *huc*, *islet1*, *oligo2*, and *gata2* in zebrafish embryonic brains (Figures S4A–S4C, and data not shown). In morphant embryos, the expression of *nestin* and *sox2* were dramatically decreased (Figures S4A andS4C, and data not shown) when we measured the zebrafish embryos at 24 hpf (hours post fertilization), 48 hpf, 50 hpf. The expression of neuron marker gene *huc*, oligodendrocyte marker gene *oligo2*, and astrocyte marker gene *gfap* were dramatically enhanced in the morphant embryos (Figures S4B andS4C, and data not shown). The increased expression of the *huc* gene and decreased expression of *sox2* gene were observed in DO MUT embryos at 48 hpf (Figures 2A–2C). Injections of DO MO did not exacerbate the phenotypes (Figures 2A–2C), demonstrating that the phenotypes produced by DO MO injection is indeed caused by targeting *dopey2* RNA. After we injected DO MO, the number of Huc positive neurons largely increased in the transgenic zebrafish embryos with Huc-GFP expression in neurons (MS1, 2 and data not shown). The same phenotypes were obtained in *dopey2* mutant zebrafish and in truncated *dopey2* RNA injected embryos (data not shown). The immunostaining of Sox2 confirmed that the number of neural cells with features of stem cells/progenitors were significantly reduced after the disruption of Dopey2 functions in the zebrafish embryos (Figures 2D, S4F, andS4G). Thus, the results indicate that Dopey2 suppresses the differentiation of neural stem cells/progenitors during the embryonic development of brains in zebrafish.

We measured the growth of neural cells by BrdU incorporation in zebrafish embryos. The number of BrdU labeled neural cells was greatly reduced in zebrafish morphant embryos (Figures S4D andS4G). The detection of H3S10p, one of the markers which labels the growing cells, showed that H3S10p positive neural cells were also dominantly reduced in zebrafish morphants and DO MUT embryos (Figures 2E, S4E, andS4G). The detection of another marker for growing cells, PCNA (data not shown), confirmed the observations that disturbed Dopey2 function caused the growing defects of neural cells seen in zebrafish embryos. Together, the results indicate that Dopey2 is responsible for the proliferation of neural stem cells and progenitors by preventing the differentiation of neural stem cells and progenitors during the embryonic neurogenesis in zebrafish.

### Dopey2 restrains the expression of Pcdh7 protein in zebrafish embryos

**Figure 3:**
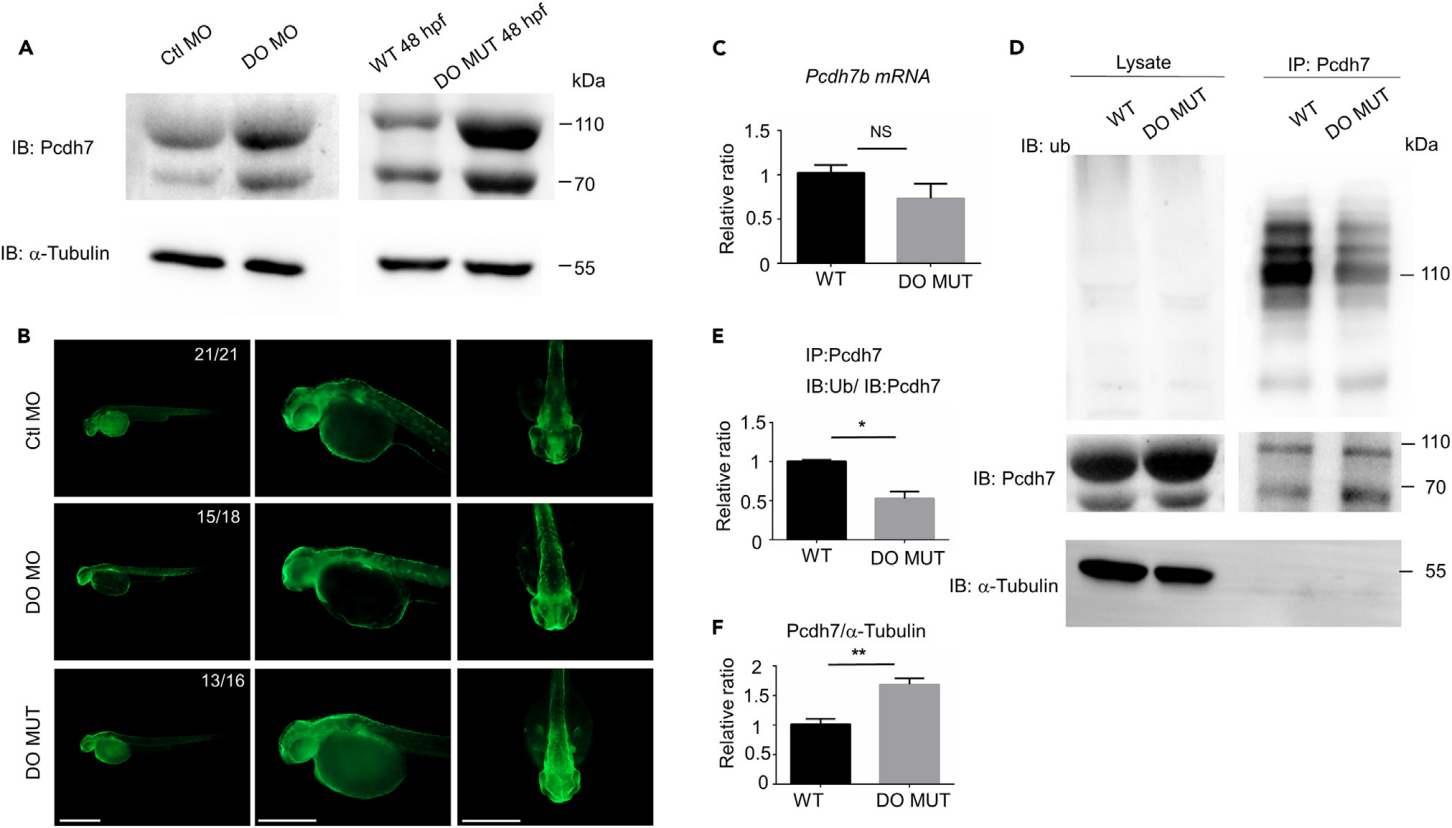
Dopey2 modulates the ubiquitination of Pcdh7b protein (A) Left panel illustrates immunoblotting detected Pcdh7 protein levels in 48 hpf WT embryos pre-injected with Ctl MO or DO MO; right panel illustrates immunoblotting detected Pcdh7 protein levels in 48 hpf WT or DO MUT embryos. Immunoblotting of α-tubulin was used as loading control. (B) Whole-mount immunofluorescence staining detected Pcdh7 protein in Ctl MO or DO MO injected WT embryos and DO MO embryos at 48 hpf. Scale bar = 500 μm. (C) Real-time fluorescence quantitative PCR analysis detected relative expression level of *pcdh7b* in WT or DO MUT embryos. (mean ± s.e.m, n = 3, Student’s *t* test: NS = not significant). (D) Co-immunoprecipitation (co-IP) analysis detected the ubiquitination of Pcdh7 in 48 hpf WT or DO MUT embryos. IP: immunoprecipitation, IB: immunoblot, Ub: anti ubiquitin antibody. (E) Quantification of immunoprecipitated Pcdh7 (DO MUT embryos) indicated a reduction in ubiquitination (mean ± s.e.m, n = 3, Student’s *t* test: ∗p < 0.05). Images were quantified by ImageJ software. (F) Quantification of immunoprecipitated Pcdh7 (DO MUT embryos) indicated Pcdh7 had an increase at protein level in lysates (mean ± s.e.m, n = 3, Student’s *t* test: ∗p < 0.05). Images were quantified by ImageJ software. Ctl MO: control MO, DO MO: *dopey2* MO, WT: wild type, DO MUT: *dopey2*mutant.

To address the mechanisms how Dopey2 modulates the proliferation of neural stem cell/progenitors, we screened protein expression patterns, mostly plasma membrane proteins, in the embryonic brains of zebrafish and identified that expression of Pcdh7, which we have previously identified is highly expressing during the differentiation programs of neural stem cells and progenitors,^21^ greatly increased in the zebrafish embryos with the dysfunction of Dopey2. Therefore, we continued to confirm Pcdh7 expression after disrupting Dopey2 functions in zebrafish embryos using an anti-human PCDH7 antibody (Figures 3A and 3B and data not shown). We found that knock down of Dopey2 expression or *dopey2* gene mutation in zebrafish embryos dramatically increased the Pcdh7 protein levels in zebrafish embryos tested by the western blotting and immunofluorescence (Figures 3A, 3B, and3F). We started to determine the relationship between Dopey2 and Pcdh7. Immunofluorescence staining showed that both proteins were distributed close to the cellular membrane but not localized together in GL251, HEK293T and U251 cells (Figure S5A). Consistently, immunoprecipitation results showed that Dopey2 was unable to pull down Pcdh7. Vice versa, Pcdh7 could not pull down Dopey2 protein (Figures S5A andS5B and data not shown). The results indicate that Dopey2 and Pcdh7 proteins are not located in the same complex. Next, we addressed how Dopey2 modulated the expression of Pcdh7 protein. We found that *pcdh7b* mRNA levels was not altered or was slightly decreased by the knocking down Dopey2 expression in zebrafish embryos (Figure 3C). It means that Dopey2 is not involved in the transcriptional regulation of *pcdh7b* gene. Ubiquitination has long been recognized as a key determinator of protein fate as this process tags proteins for proteasomal degradation. Hence, we used an immunoprecipitation approach to test ubiquitin labeled Pcdh7 protein in zebrafish embryos. Indeed, Pcdh7 protein was labeled by ubiquitin and was involved in the proteasomal degradation by ubiquitination (Figure 3D). After *dopey2* genetic mutation, ubiquitin labeled Pcdh7 was greatly decreased in zebrafish embryos (Figures 3D and 3E), indicating that Dopey2 is involved in the pathways through which Pcdh7 is degraded by the ubiquitin proteasome system.

### Pcdh7 constrains the expression of *dopey*2 mRNA in zebrafish embryos

**Figure 4:**
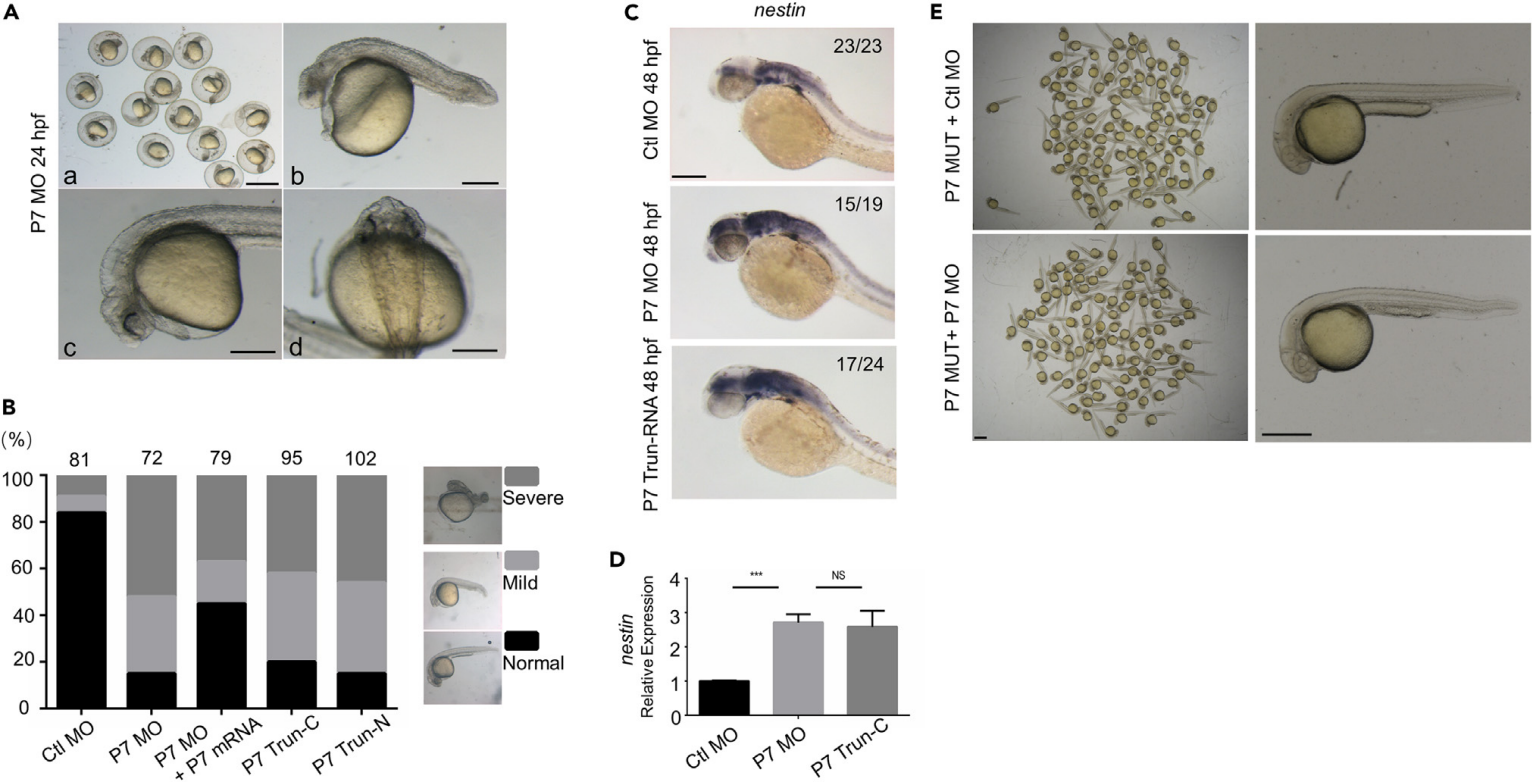
Knocking down *pcdh7b* caused defects in zebrafish embryos (A) Phenotypes of P7 MO injected zebrafish embryos at 24 hpf. a, Scale bar = 1 mm; b-d, Scale bar = 100 μm. (B) Left panel indicates ratio of phenotypes in each group injected with Ctl MO (2 ng/μL, n = 81), P7 MO (2 ng/μL, n = 72), P7 MO + P7 mRNA (P7 MO 2 ng/μL +P7 mRNA 60 ng/μL, n = 79), P7 TRUN-C (120 ng/μL, n = 93), P7 TRUN-N (120 ng/μL, n = 102). Right panel shows different phenotype of MO injected embryos at 24 hpf. (C) *In situ* hybridization analysis detected *nestin* gene in embryos pre-injected with Ctl MO, P7 MO or P7 TRUN-RNA. Scale bar = 200 μm. (D) Real-time fluorescence quantitative PCR analysis showed the relative expression *nestin* genes in embryos at 48 hpf, embryos were pre-injected with Ctl MO, P7 MO or P7 TRUN-C (mean ± s.e.m, n = 3, Student’s *t* test: ∗∗∗p < 0.001, NS = not significant). (E) Phenotypes of P7 MUT injected with Ctl MO or P7 MO zebrafish embryos at 24 hpf. Left, Scale bar = 1 mm; Right, Scale bar = 500 μm. Ctl MO: control MO, P7 MO: *pcdh7b* MO, P7 mRNA: *pcdh7b* mRNA, P7 TRUN: *pcdh7b* truncated mRNA.

To figure out which zebrafish paralogous *pcdh7* gene is conserved to human *PCDH7* (Figure S6A), we screened *pcdh7a* MOs and *pcdh7b* MOs in zebrafish embryos and identified that zebrafish *pcdh7b* MO (P7 MO) knocked down the protein expression detected by the anti-human PCDH7 antibody (Figures S7A andS8B). Consistently, we noticed that there were no observable alterations in brain development or neural marker expression following the knockdown of *pcdh7a* MO in zebrafish embryos (Figures S6B andS6C). Thus, human PCDH7 protein is the conserved homologue of zebrafish Pcdh7b. To tested whether the expression of Dopey2 was altered by Pcdh7b, we used antisense morpholino oligonucleotides complementary to the slicing site (P7 MO) and translation start site (P7 MO-AUG) of zebrafish *pcdh7b* RNA as well as truncated RNAs of *pcdh7b*N-terminal and C-terminal deleted mutants and the *pcdh7b* mutant zebrafish (P7 MUT) generated by CRISPR/Cas9 system to determine the relationship between Dopey2 and Pcdh7 in zebrafish embryos (Figures S6D, S7B, andS8A). When P7 MO was used to target the splice site, it was found that *pcdh7b* RNA was not properly cleaved (Figure S7A), resulting in a decrease in Pcdh7 protein level (Figure S8B) and neural defective phenotypes in zebrafish embryos (Figures 4A–4D). Expression of truncated Pcdh7 was also found to cause neural defective phenotypes in zebrafish embryos (Figures 4B–4D). By injecting P7 MO-AUG, which targets the translational start site of *pcdh7b* mRNA, and a P7 MO-AUG target site-eGFP recombinant plasmid into zebrafish embryos, it was shown that P7 MO-AUG effectively inhibited the expression of eGFP (Figure S7B) and caused neural defective phenotypes in zebrafish embryos (data not shown). In P7 MUT embryos, mutations of *pcdh7b* gene diminished the expression of Pcdh7 protein in zebrafish embryos (Figure S8B). However, it was observed that mutations of *pcdh7b* gene did not exhibit any observable changes in phenotypes in zebrafish embryos (Figures 4E, S8C, and S8D). In addition, the injection of P7 MO into P7 MUT embryos failed to produce any observably phenotypic changes in zebrafish embryos (Figures 4E, S8C, and S8D). Previous studies have discovered that mutations containing PTCs can result in transcriptional compensation, and a genetic compensation effect is concealing the effects of the morpholino reactions.^23^^,^^24^^,^^25^ The facts that injections of P7 MO into P7 MUT embryos do not cause any defective phenotypes indicate that mutations of *pcdh7b* gene conceal the P7 MO effects in P7 MUT zebrafish embryos, confirming that zebrafish embryonic phenotypes are caused by P7 MO specifically targeting *pcdh7b* RNA rather than other RNAs. All tests of the approaches demonstrate that *pcdh7* morpholino oligonucleotides show nicely the efficiency and credibility to manipulate zebrafish embryos.

**Figure 5:**
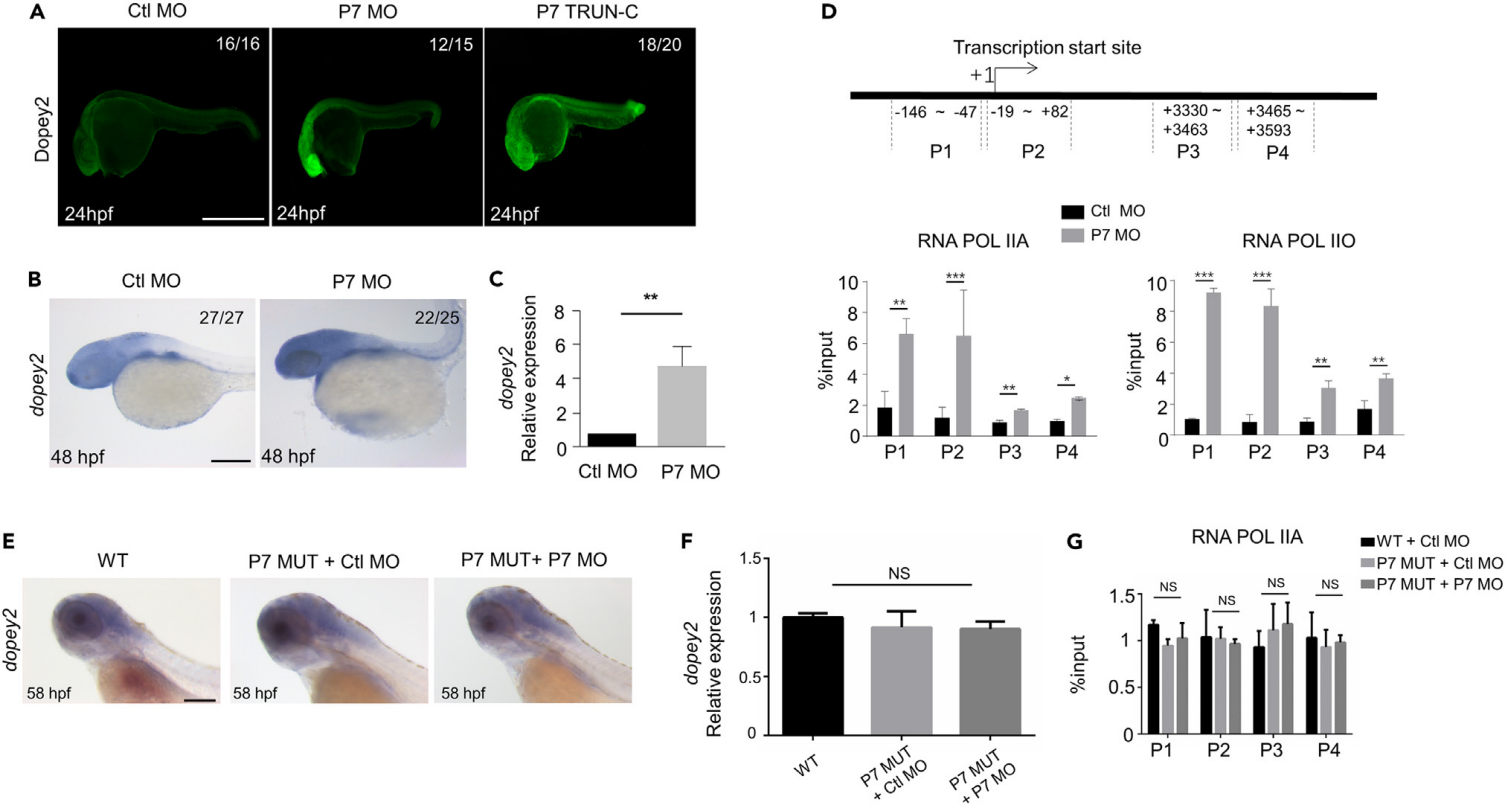
Pcdh7b regulates the transcription of *dopey2* in zebrafish embryos (A) Whole-mount immunofluorescence staining detected Dopey2 protein in Ctl MO, P7 MO and P7 TRUN injected embryos at 24 hpf. Scale bar = 500 μm. (B) *In situ* hybridization analysis detected the expression of *dopey2* in 48 hpf embryos pre-inject Ctl MO or P7 MO. Scale bar = 200 μm. (C) Real-time fluorescence quantitative PCR analysis detected the relative expression level of *dopey2* gene in 48 hpf embryos pre-injected with Ctl MO or P7 MO (mean ± s.e.m, n = 3, Student’s *t* test: ∗∗p < 0.01). (D) The top panel represents schematic diagram of *dopey2* gene primers for ChIP analysis. The bottom panel showed ChIP analysis results of 48 hpf embryos pre-inject with Ctl MO or P7 MO (mean ± s.e.m, n = 3, Student’s *t* test: ∗∗∗p < 0.001, ∗∗p < 0.01, ∗p < 0.05). (E) *In situ* hybridization analysis detected the expression of *dopey2* in 58 hpf WT embryos or P7 MUT embryos pre-injected with Ctl MO or P7 MO. Scale bar = 200 μm. (F) Real-time fluorescence quantitative PCR analysis detected the relative expression level of *dopey2* gene in 48 hpf WT embryos or P7 MUT embryos pre-injected with P7 MO (mean ± s.e.m, n = 3, Student’s *t* test: NS = not significant). (G) ChIP analysis results of 48 hpf WT embryos or P7 MUT embryos pre-injected with Ctl MO or P7 MO (mean ± s.e.m, n = 3, Student’s *t* test: NS = not significant). Ctl MO: control MO, P7 MO: *pcdh7b* MO, RNA POL IIA: RNA polymerase II with an unphosphorylated CTD, RNA POL IIO: RNA polymerase II with a phosphorylated CTD, WT: wild type, P7 MUT: *pcdh7b*mutant.

We injected *pcdh7* morpholino oligonucleotides and mRNA of truncated Pcdh7 mutants into zebrafish embryos to detect *dopey2* mRNA expression. The results showed that levels of Dopey2 were dramatically increased in the zebrafish embryos by knocking down Pcdh7 or after expression of the truncated *pcdh7* RNA (Figures 5A–5C), indicating that Pcdh7 participate in the pathway that regulates the generations of *dopey2* mRNA. We used chromatin immunoprecipitation (ChIP) assay to test whether Pcdh7 participates in the pathway that regulates the transcription of *dopey2* gene. RNA polymerase II (Pol II) transcribes all protein-coding genes. The C-terminal domain (CTD) of the largest subunit of RNA polymerase II consists of multiple heptad repeats (consensus Tyr1–Ser2–Pro3–Thr4–Ser5–Pro6–Ser7). In general, after the formation of the pre-initiation complex (PIC) at the promoter of a gene, RNA polymerase II with an unphosphorylated CTD, which is detected by the antibody 8WG16, initiates the transcription of a gene.^26^ Then, the CTD is phosphorylated on Ser5, which is detected by an anti-RNA polymerase II CTD repeat YSPTSPS (phospho S5) antibody, for the elongation of gene transcription.^27^^,^^28^ We performed ChIP to measure the RNAP II states along the *dopey2* gene. The results displayed that Pcdh7 knockdown dominantly increased the number of RNAP II with an unphosphorylated CTD and with Ser5 phosphorylated CTD localized within the promoter region and transcription initiating region of *dopey2* gene (Figure 5D). This phenomenon was also observed when morpholino targeting the translation start site was injected into embryos (Figures S7C and S7D). We observed that there were no significant changes in *dopey2* gene expression or transcription in P7 MUT embryos (Figures 5E–5G). In addition, injections of P7 MO into P7 MUT embryos did not alter *dopey2* expression or transcription activation by RNAP II loading, confirming that P7 MO affects *dopey2* transcription by targeting the expression of *pcdh7b* gene (Figures 5E–5G). Together, the results indicate that Pcdh7 is involved in the pathway to restrict the transcription of *dopey2* gene in zebrafish embryos.

### Pcdh7 is responsible for the differentiation programs of embryonic stem cells and progenitors

**Figure 6:**
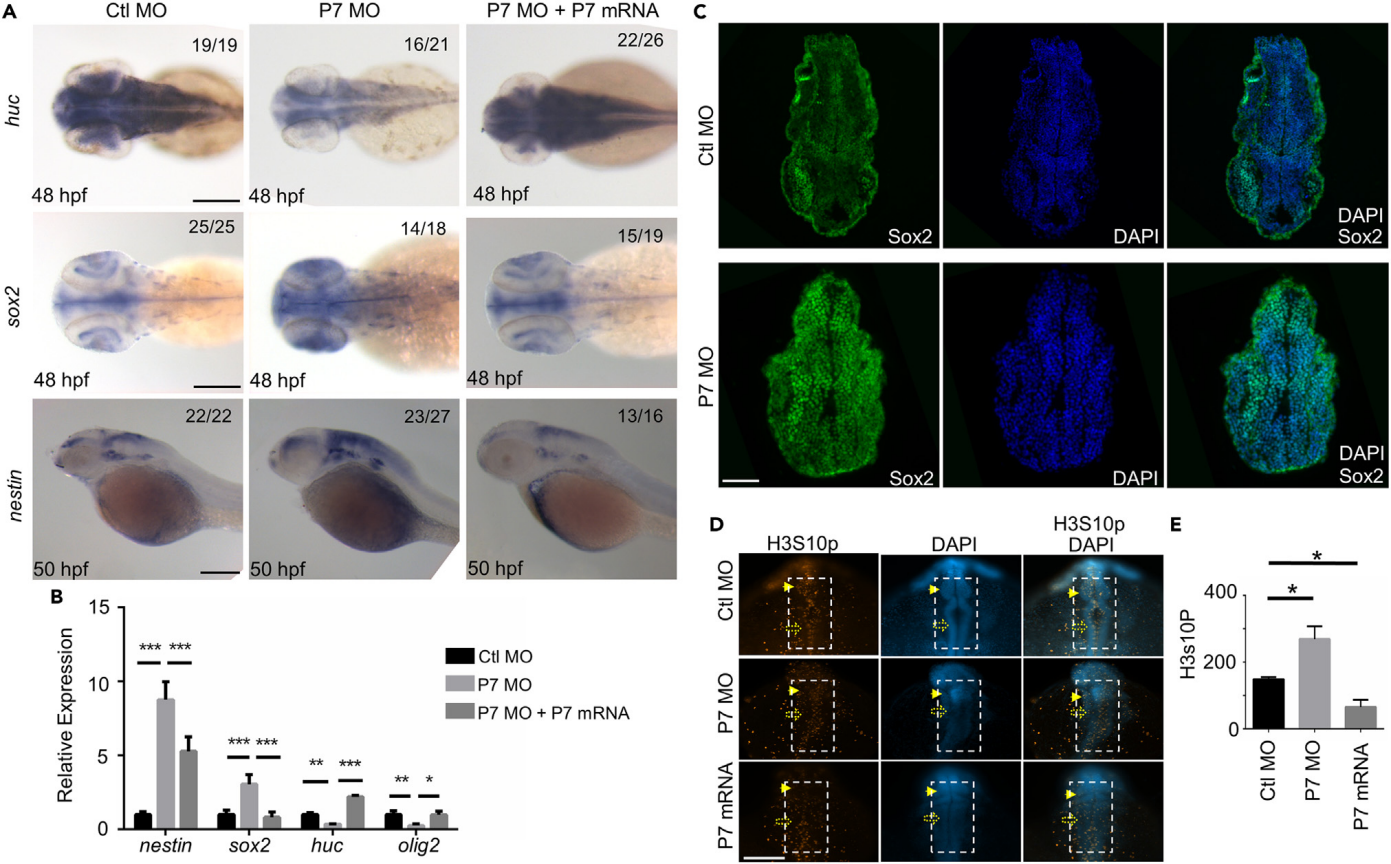
Pcdh7b modulates the differentiation of neural stem cells/progenitors and inhibits the proliferation of neural stem cells/progenitors in zebrafish embryonic brains (A) *In situ* hybridization analysis detected the expression of *nestin*, *sox2*, and *huc* genes. Emryos were at 48 hpf, 50 hpf, pre-injected with Ctl MO, P7 MO and P7 MO + P7 mRNA. Scale bar = 200 μm. (B) Real-time fluorescence quantitative PCR analysis showed the relative expression level of *nestin*, *sox2*, *huc* and *olig2* gene at 48 hpf in embryos pre-injected with Ctl MO, P7 MO and P7 MO + P7 mRNA (mean ± s.e.m, n = 3, Student’s *t* test: ∗∗∗p < 0.001, ∗∗p < 0.01, ∗p < 0.05). (C) Representative immunofluorescence staining detected Sox2 in the frozen sections of 24 hpf embryos pre-injected with Ctl MO or P7 MO. Scale bar = 50 μm. (D) Whole-mount immunofluorescence staining for H3S10P in Ctl MO, P7 MO and P7 mRNA pre-injected embryos at 24 hpf. Scale bar = 100 μm. (E) Statistical analysis of the number of H3S10P-positive cells in embryos pre-injected with Ctl MO, P7 MO and P7 mRNA. The number in the dotted box region in (A) was counted. For each group, 6 embryos were scored (mean ± s.e.m, n = 3, Student’s *t* test: ∗p < 0.05). Images were quantified by ImageJ software. Ctl MO: control MO, P7 MO: *pcdh7b* MO, P7 mRNA: *pcdh7b* mRNA.

To test Pcdh7b functions during the development programs of zebrafish embryos, we firstly analyzed temporal and spatial expression patterns of *pcdh7b* gene. The results showed that *pcdh7b* gene was zygotically expressed in zebrafish embryos (Figures S9A andS9B). The embryonic neural cells dominantly carried highly *pcdh7b* expressing mRNA levels (Figure S9C). Because of the genetic compensation effects in zebrafish embryos with *pcdh7* gene mutants and the *pcdh7* morpholino oligonucleotides specifically targeting to the *pcdh7b* RNA, we used *pcdh7* morpholino oligonucleotides to perform experiments in detail and P7 MUT zebrafish to confirm the specificities of results in zebrafish embryos. In zebrafish P7 MO morphant embryos, the expression of *nestin* and *sox2* were dramatically increased when we measured the zebrafish embryos at the stages of 48 hpf, 50 hpf (Figures 6A and 6B). The immunostaining of Sox2 confirmed that the neural cells with features of stem cells/progenitors were significantly enhanced after the disruption of Pcdh7b functions in the zebrafish embryos (Figure 6C). The expression of neuron marker gene *huc*, oligodendrocyte marker gene *oligo2*, and astrocyte marker gene *gfap* were dramatically reduced in the morphant embryos (Figures 6A, 6B, and S9D, and data not shown). Similarly, embryos injected with P7 MO-AUG at 48 hpf led to an elevation of *sox2* expression and decreased *huc* expression in the embryos (Figure S7E). Injections *of pcdh7* morpholino oligonucleotides into P7 MUT zebrafish embryos did not cause any phenotypes, confirming again the specificities of neural phenotypes caused by *pcdh7* morpholino oligonucleotides (Figures S8C andS8D). After we injected P7 MO, the distribution of Huc positive neurons were disturbed and greatly decreased in the transgenic zebrafish embryos with Huc-GFP expression in neurons (data not shown, MS1, 4). The detection of H3S10p showed that the number of H3S10p positive neural cells were dominantly increased in zebrafish P7 MO morphant embryos (Figures 6D and 6E). Pcdh7b overexpression dramatically decreased the H3S10p positive cells in embryonic brains (Figures 6D and 6E). Together, the results indicate that Pcdh7b is responsible for the differentiation of neural stem cells and progenitors and prevents the proliferation of neural stem cells and progenitors during embryonic neurogenesis, just the opposite of Dopey2 functions during embryonic neurogenesis.

### Dopey2 and Pcdh7 are required to modulate the size and architecture of developmental brains in zebrafish embryos

**Figure 7:**
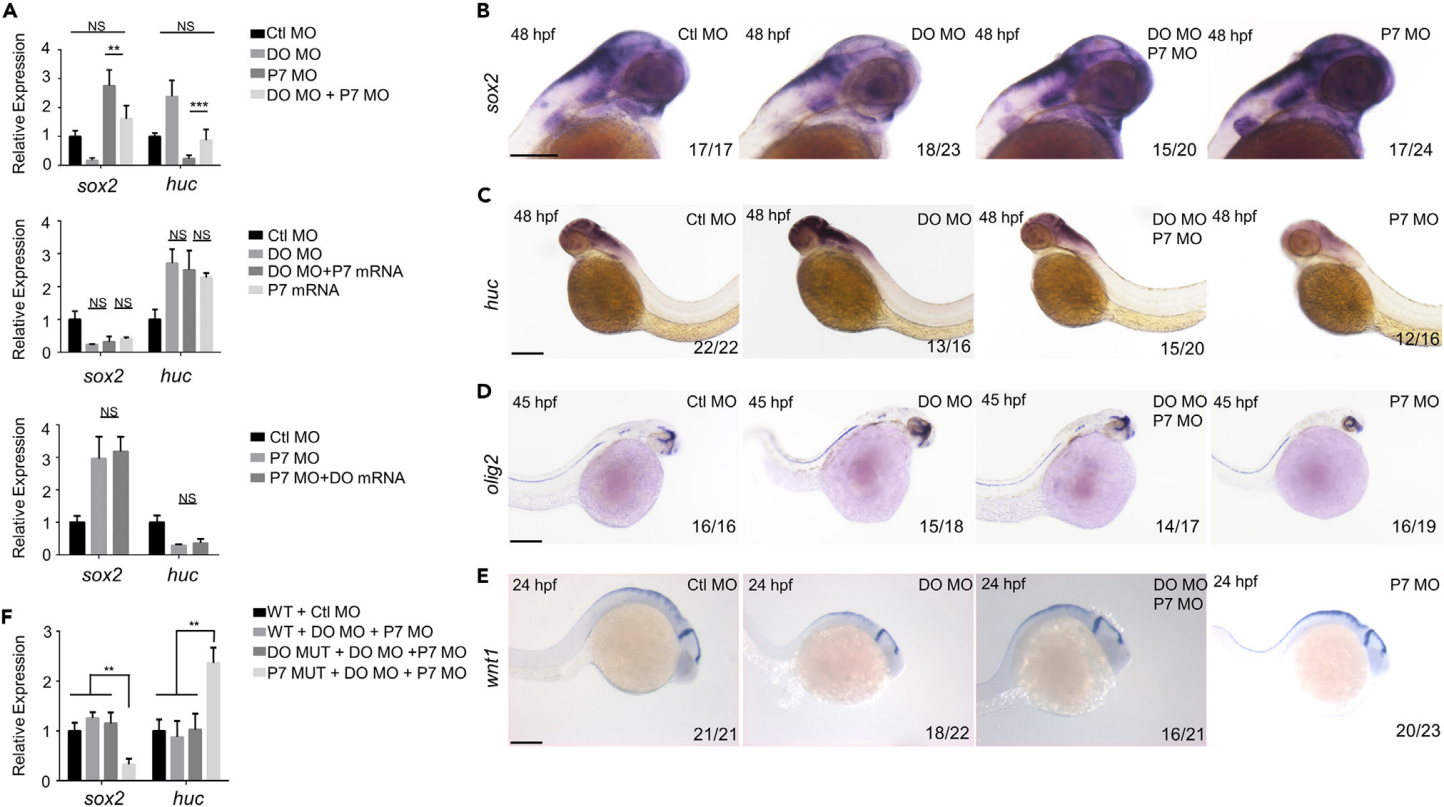
Dopey2 and Pcdh7b complement the development and arrangement of neural cells in zebrafish embryonic brains (A) Real-time fluorescence quantitative PCR analysis shows the relative expression level of *sox2*, *huc* at 48 hpf in embryos pre-inject with Ctl MO, DO MO, P7 MO, DO MO + P7 MO, DO MO + P7 mRNA, P7 mRNA, and P7 MO + DO mRNA (mean ± s.e.m, n = 3, Student’s *t* test: ∗∗∗p < 0.001, ∗∗p < 0.01, ∗p < 0.05). (B–E) ISH analysis of *sox2*, *huc*, *olig2* and *wnt1* for embryos at 48 hpf, 45 hpf and 24 hpf pre-injected with Ctl MO, DO MO, P7 MO and DO MO + P7 MO. Scale bar = 200 μm. (F) Real-time fluorescence quantitative PCR analysis shows the relative expression level of *sox2*, *huc* at 48 hpf in WT, DO MUT or P7 MUT embryos pre-inject with Ctl MO, DO MO + P7 MO (mean ± s.e.m, n = 3, Student’s *t* test: ∗∗p < 0.01). Ctl MO: control MO, DO MO: *dopey2* MO, DO mRNA: *dopey2* mRNA, P7 MO: *pcdh7b* MO, P7 mRNA: *pcdh7b* mRNA, DO MUT: *dopey2*mutant, P7 MUT: *pcdh7b*mutant.

To determine the functional relationship between Dopey2 and Pcdh7, we coinjected DO MO and P7 MO into one-cell embryos. The results turned out that the expression of *sox2* and *nestin* (data not shown) was comparable to ones in the embryos injected with the Ctl MO (Figures 7A and 7B). DO MO injections decreased the enhanced expression of *sox2*, *nestin* and *wnt1* by P7 MO (Figures 7A, 7B, and 7E). On the other hand, P7 MO injection reduced the higher expression of *huc, gfap* and *oligo2* caused by DO MO in zebrafish embryos (Figures 7A, 7C, and 7D, and data not shown). Furthermore, injection of *pcdh7* mRNA was unable to increase the enhanced expression of *huc, gfap* and *oligo2* induced by DO MO in zebrafish embryos (Figure 7A, and data not shown). In return, the injection of *dopey2* mRNA also could not alter the expression of *sox2* and *nestin* induced by P7 MO (Figure 7A). The simultaneous injection of DO MO and P7 MO into DO MUT embryos was found to ease off the effects of increased sox2 expression and decreased huc expression caused by P7 MO (Figures 7A–7C). On the other hand, the simultaneous injection of DO MO and P7 MO into P7 MUT embryos did not mitigate the phenotypes of decreased sox2 expression and increased huc expression caused by DO MO (Figure 7F). The findings indicate that P7 MO can reverse the effects of loss of Dopey2 function in the DO MUT and that the addition of DO MO does not alter this outcome. However, in the P7 MUT, the presence of a genetic compensation mechanism results in decreased sox2 and increased huc following DO MO injection, which confirms that the phenotype caused by the combination of DO MO and P7 MO is indeed because of the targeted mechanism. Together, the examinations suggest that Dopey2 and Pcdh7 are involved in a similar way to modulate the proliferation and differentiation of neural stem cell/progenitors during the development of embryonic brains.

**Figure 8:**
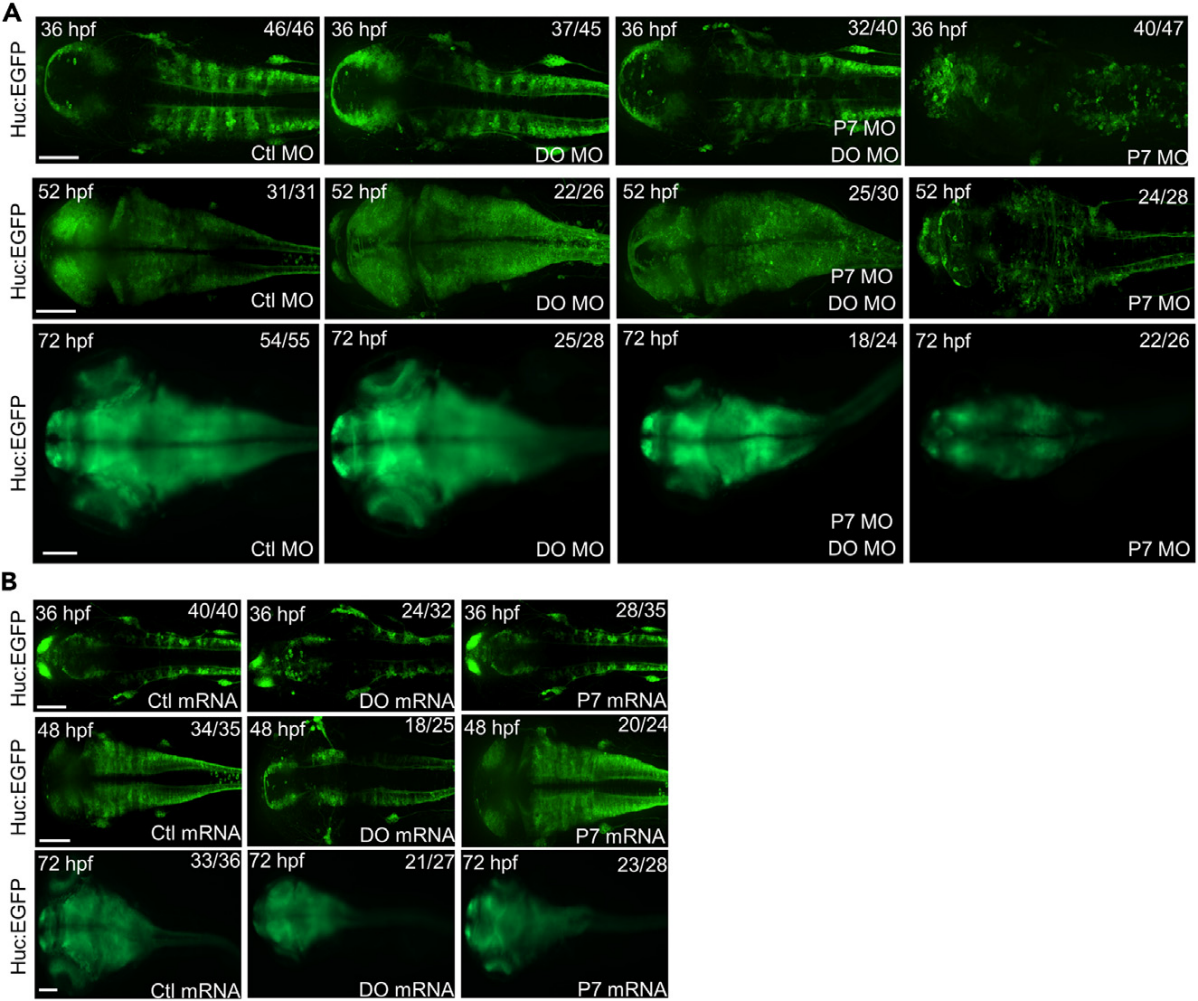
Dopey2 and Pcdh7b orchestrates the development and arrangement of neural cells in zebrafish embryonic brains (A) Embryos of *huc*:EGFP zebrafish pre-injected with Ctl MO, DO MO, P7 MO and DO MO + P7 MO at 36 hpf, 52 hpf and 72 hpf shows Huc in green fluorescence. Scale bar = 100 μm. (B) Embryos of *huc*:EGFP zebrafish pre-injected with Ctl mRNA, DO mRNA and P7 mRNA at 36 hpf, 48 hpf and 72 hpf showed Huc in green fluorescence. Scale bar = 100 μm.

We visualized the neurons and structures of embryonic brains in the transgenic zebrafish embryos with Huc-GFP expression in neurons by co-injection of DO MO and P7 MO (Figure 8A, Videos S1, S2, S3, and S4). By knocking down both the expression of Dopey2 and Pcdh7b in embryonic brains, we were able to restore the arrangement of neurons, which was disturbed by the disruption of Pcdh7 function. However, the brain did not reach a similar size when compared to brains in the zebrafish embryos injected with Ctl MO. After Dopey2 knockdown, neuron formation in the brains of zebrafish embryos was highly increased. The sizes of brains were larger at 72 hpf, the end of the embryonic development in zebrafish, in comparison to the control brains. Knocking down Pcdh7b largely reduced neuron formation and affected neuron arrangements in zebrafish embryonic brains, resulted in the smaller size of brains at 72 hpf of zebrafish embryos. Overexpression of Dopey2 or Pcdh7b caused the smaller size brains at 72 hpf (Figure 8B, Videos S5, S6, and S7). Consistent with the results, overexpression of Dopey2 decreased neuron formation during the development progress detected at 18 hpf (data not shown), 36 hpf, 48 hpf, and 72 hpf (Figure 8B, Videos S5, S6, and S7). However, overexpression of Pcdh7b increased the neuron formation during the development progress detected at 18 hpf (data not shown), 36 hpf, 48 hpf (Figure 8B). At 72 hpf, overexpression of Pcdh7b caused a smaller size of zebrafish brains (Figure 8B, Videos S5, S6, and S7). Taken together, we observed that Dopey2 maintains the proliferation of neural stem cells and progenitors and suppresses the neural differentiation, modulates the number of neural cells in embryonic brain. On the other hand, Pcdh7 is responsible for the differentiation of neural stem cells and progenitors and the arrangements of neural cell and inhibits the neural proliferation during the embryonic neurogenesis. Both Dopey2 and Pcdh7 proteins are required for the proper development of embryonic brain in zebrafish.

Video S1. 52 hpf Ctl MO_Trim, related to Figure 8

Video S2 52 hpf DO MO_Trim, related to Figure 8

Video S3. 52 hpf DO MO P7 MO_Trim, related to Figure 8

Video S4. 52 hpf P7 MO_Trim, related to Figure 8

Video S5. 48 hpf Ctl mRNA_Trim, related to Figure 8

Video S6. 48 hpf dopey2 mRNA_Trim, related to Figure 8

Video S7. 48 hpf pcdh7b mRNA_Trim, related to Figure 8

## Discussion

Cellular proliferation versus differentiation of neural stem cells and progenitors has been largely investigated. Initially, the embryonic brain begins with primitive neural precursors, neuroepithelial cells, which gives rise to all neurons and macroglia in the brain.^29^^,^^30^ Then, neuroepithelial cells begin to differentiate into the population of more fate-restricted stem cells/progenitors that signify common stem cells/progenitors for neurons, astrocytes, and oligodendrocytes.^31^^,^^32^ The common stem cells/progenitors asymmetrically generate neural cells or produce intermediate progenitor populations that transiently increase the progenitor pool before terminally differentiating into specific types of neuronal and glial cells, each with their precise function.^33^ To effectively perform the complicated processes in the developmental brain, intrinsic (e.g., transcription factors) and extrinsic (environmental signals) factors synchronize the determination by neural progenitors to continue to undergo proliferation or to differentiation to produce the populations of neurons and glia cells.^34^^,^^35^^,^^36^ Appropriate numbers of inhibitory and excitatory neurons, oligodendrocytes, and astrocytes are required for normal neural functions in animals. Any imbalance in the population of specific neurons, astrocytes, and oligodendrocytes can cause neurophysiological, neuropsychological and intellectual disorders.^37^ Studies in the embryonic brain of model animals have identified many factors leading to the understanding of the brain development. It has been revealed that the neurons, astrocytes, and oligodendrocytes that compose the mature brain are generated sequentially from neural stem cell/progenitor pools. The balance of proliferation and differentiation of neural stem and progenitor cells during embryonic development is a causal factor to determine the architecture and the size of brains.^38^^,^^39^^,^^40^ The mechanisms how the balance of proliferation and differentiation of neural stem and progenitor cells is maintained remain unknown. In present study, we demonstrate two molecules, which restrain their expression mutually, to balance proliferation and differentiation of neural stem cells and progenitors and to modulate the cellular number and arrangements for the proper size of embryonic brain. The finding provides a novel way to understand how embryonic brain is constituted.

We demonstrated that Dopey2 controls the neural development and brain maturation by maintaining the proliferation of neural stem cells and progenitors and inhibits the neural differentiation during embryonic neurogenesis. Our results indicate when Dopey2 highly expresses in the brain, neural differentiation is suppressed in certain regions, resulting the defective brain maturation, fewer neurons, and lower neuronal density. The appearance phenocopied the neuromorphological features in DS patients. We have also identified that Pcdh7 is responsible for the differentiation of neural stem cells and progenitor and modulates the neural architecture during embryonic development of zebrafish brains. Increased expression of Pcdh7 reduces the proliferation of neural stem cells and progenitors and enhances the neural differentiations. Disruption or increased expression of Pcdh7b functions causes neural defects during embryonic brains. The phenotypes we saw might explain the developmental defects in the brains of patients with Rett syndrome and *MECP2* duplication syndrome. Our results indicate both Dopey2 and Pcdh7 are required to balance the proliferation and differentiation of neural stem cells and progenitors during embryonic neurogenesis to generate proper sizes and architectures of brains.

### Limitations of the study

We showed that Dopey2 and Pcdh7 mutually restrict their expression through different ways. Previous data show that DOPEY2 is involved in the sorting nexin-3 (SNX3) containing complex.^41^^,^^42^ SNX3 containing complex retrieves Wntless (Wls) from the early endosome back to the Golgi complex, prevents Wls lysosomal degradation and allows Wls to undergo COPI-mediated retrograde transport back to the ER. DOPEY2-associated complex displays an evolutionary conserved endosome-associated membrane re-modeling complex. *In vivo* suppression of DOPEY2 leads to enhanced lysosomal degradation of Wntless.^41^^,^^42^ The observations suggest that DOPEY2 prevents the lysosomal degradation of proteins. In our present work, we identify that DOPEY2 does not locate with PCDH7 in cells, excluding the hypothesis that lysosomal degradation of PCDH7 mediated directly by DOPEY2. In contrast, Dopey2 is able to modulate the ubiquitination of Pcdh7 and subjects the Pcdh7 protein to the degradation of ubiquitin proteasome system. In present works, we failed to show how Dopey2 is involved in the pathway of ubiquitin proteasome system. The questions need to be further addressed. We showed that Pcdh7 regulates *dopey2* RNA expression through modulating RNA polymerase II loading to the promoter of *dopey2* gene*.* After transcription initiates, Pcdh7 is also able to keep the RNA polymerase II for transcription elongation along the *dopey2* gene. Many processes and protein complexes, for instance, complexes for DNA and histone modifications, chromatin remodeling complexes, mediator complexes, pre-initiation complex, or DSIF complex, are involved in RNA polymerase II loading to a gene promoter and for the transcription elongation of RNA polymerase II.^26^^,^^28^^,^^43^ In our present work, we did not figure out how PCDH7, a membrane protein, regulates the nuclear affair for the transcription of a specific gene. Future, more in depth approaches are required to identify the pathways and mechanisms as to how the transcription of a specific gene is regulated by PCDH7.

## Data and materials availability

All data are available in the main text or the supplemental information.

## STAR★Methods

### Key resources table

**Table:** 

REAGENT or RESOURCE	SOURCE	IDENTIFIER
**Antibodies**		

Anti-GFAP	Thermo Fisher	Cat#85261
Anti-MAP2	Santa Cruz	Cat#sc-7442
Anti-MBP	Santa Cruz	Cat# sc-27152
Anti-TuJ-1	Novus	Cat# NB100-1612
Anti-p-histone H3 (S10)	Millipore	Cat#04-817
Anti-Sox2	Abcam	Cat# ab97959
Anti-Dopey2	HUABIO	Cat#0903-3
Anti-BrdU	Sigma	Cat# ab6326
Anti-ɑ-tublin	Abcam	Cat# ab15246
Anti-Pcdh7	Abcam	Cat# ab139274

**Chemicals, peptides, and recombinant proteins**		

TRIzol	Molecular Research Center, Inc.	TR118
Fetal Bovine Serum	Hyclone	SH30396
PTU	Sigma	2954-52-1
Methanol	CHRON CHEMICALS	67-56-1

**Critical commercial assays**		

NucleoSpin RNA Plus	MACHEREY-NAGEL	740984.50
mMESSAGE mMACHINE T7 Transcription Kit	Ambion	AM1344
Q5 High-Fidelity DNA Polymerase	NEB	M0491
FastStart Essential DNA Green Master	Roche	06402712001
PrimeScrip RT reagent Kit	Takara	RR037
ChIP Assay Kit	Beyotime	P2078
Universal DNA Purification Kit	TIANGEN	4992197
T7 RNA Polymerase	Promega	P2075
SP6 RNA Polymerase	Promega	P1085

**Experimental models: Organisms/strains**		

Mouse: BALB/c	DOSSY	BALB/c
Zebrafish AB	CZRC	CZ1
Zebrafish AB: Tg(*huc*:EGFP)	CZRC	CZ160
Zebrafish AB: CRISPR/Cas9 *dopey2*	This paper	N/A
Zebrafish AB: CRISPR/Cas9 *pcdh7b*	This paper	N/A

**Software and algorithms**		

ImageJ Software	Wayne Rasband (NIH)	N/A
Prism 5	GraphPad Inc.	N/A

### Resource availability

#### Lead contact

Further information and requests for resources and reagents should be directed to the lead contact: Xianming Mo (xmingmo@scu.edu.cn).

#### Materials availability

Zebrafish mutants generated in this study are available from lead contact upon request.

### Experimental model and subject details

#### Zebrafish maintenance

We used AB lines and Tg (*huc*:EGFP) lines to conduct experiments. Adult zebrafish (*Danio rerio*) were raised and maintained according to standard procedures. Embryos were placed in 28.5°C incubator and staged according to standard procedures. All experiments involving the use of animals were conducted in compliance with the approved guidelines. The animal protocols were approved by the Animal Care and Use Committee of West China Hospital, Sichuan University, China.

#### Establishmen of the mutant zebrafish line

The mutant zebrafish line was generated by CRISPR-Cas9 system. Optimized guide RNA was designed by CHOPCHOP and Benchling. The target sequences of Dopey2 gene in exon1 is 5′-GGCCGATCTCATCTCCTCAC-3′. The target sequences of Pcdh7b gene in exon1 is 5′-GGTAATGTAGCCGCGGACCT-3′. One-cell stage of wild-type zebrafish embryos were microinjected with 1nL sgRNA and Cas9 protein (GenScript) containing nuclear localization signal (NLS) (final concentrations is 250 ng/μL sgRNA and 150 ng/μL NLS-Cas9). After initial screening by T7EⅠ endonuclease (NEB) and Sanger sequencing, the F0 generation was crossed with wild-type zebrafish to obtain the F1 generation. Until they have grown to 1.5 months, the genotyping of each F1 were performed to obtain F1 mutants carrying frameshift mutations. The homozygous mutant F2 was obtained by mating heterozygous F1 fish with the same mutation type. The primers used to detect *dopey2* genotype were 5′-GACTGGCAGAATAACCTACA-3' (upper primer) and 5′-TTACGGCAATAATTGTTTCG-3′ (lower primer). The primers used to detect the genotype of *pcdh7b* were 5′-TATGAGGACTACAGGCATTG-3' (upper primer) and 5′-AGTGGGCAGGAGATAAAG-3′ (lower primer).

### Method details

#### Production of mouse monoclonal antibodies against mouse embryonic stem cells and identification of surface antigens

The Mouse embryonic stem cells cell (mES) lines D3 (ATCC, http://www.atcc.org/) and J2 were cultured on mitomycin C-treated mouse embryonic fibroblasts (MEFs). The mouse embryonic carcinoma (EC) cell line F9 (ATCC, http://www.atcc.org/) was cultured in Dulbecco’s Modified Eagle’s Medium (DMEM, Gibco) supplemented with 12% fetal bovine serum (FBS, Hyclone) at 37°C in 5% CO_2_. The procedures to produce mouse monoclonal antibodies against mouse embryonic stem cells and to identify surface antigens of mouse embryonic stem cells were performed. Briefly, mES D3 cells were injected into two-monthsold BALB/c mice for each immunization. Subsequent boosts were done using mES cell suspension with an interval of two weeks between each immunization. One week after the fourth immunization, serum from the immunized mice was collected and tested for immunocytochemical staining of mES cells. Two weeks after the fourth immunization, the mice were injected intravenously with mES cells to boost the immunization. The mice were sacrificed three days after the last boost, and spleen cells were isolated and used to produce hybridomas. Supernatants from the hybridoma cultures were collected. mES cells were cultured and then stained with hybridoma supernatants and a FITC conjugated goat anti-mouse secondary antibody. Positive clones were subcloned by limiting dilution in 96-well plates. The antigens were identified by hybridoma supernatant and Protein A-conjugated resin, following liquid chromatography– tandem mass spectrometry (LC–MS/MS). The membrane proteins of mouse ES cells were detected by immunofluorescence approaches and flow cytometer using the hybridoma supernatants.

#### Neural colony-forming cell assay

We based our assay on the Neural colony-forming cell (NCFC) assay and made minor modifications. To summarize, FACS was performed after NSC cells were obtained from E14 mouse embryo. 1000 cells were seeded in each 10 cm cell culture dish and cultured in semi-solid NSC proliferation medium containing 20 ng/μL EGF and bGFG to form primary neurospheres. After 14 days, individual clones (>50 μm in diameter) were picked and digested into single cells to form secondary neurospheres. After 10 days, each secondary neurosphere (>30 μm in diameter) was individually picked to form a single cell. Cells derived from each neurosphere were split into two wells of a 48-well plate and cultured in differentiation medium for 10 days. Then, multi-label immunofluorescence staining was performed according to the standard procedures, and the following antibodies were used: GFAP (Thermo Fisher, 85261, 1:800 dilution), MAP2 (Santa Cruz, sc-7442, 1:500 dilution), MBP (Santa Cruz, sc-271524, 1:500 dilution), TuJ-1 (Novus, NB100-1612, 1:1000 dilution).

#### RNA extraction and purification

Zebrafish embryos were collected at appropriate time and frozen with liquid nitrogen. Fresh TRIzol Reagent (Molecular Research Center, Inc.) was added to the sample which is then mechanically disrupted for 5 min. Chloroform was added for phase separation and the aqueous phase is mixed with isopropyl alcohol. After centrifugation, the RNA extracted is washed with 75% ethanol, and dried completely. It is then dissolved with RNAase-free ddH_2_O. For mRNA synthesized from linearized plasmid or sgRNA, we used an RNA purification kit (MACHEREY-NAGEL) to obtain RNA prepared for injection. RNA concentration and purity were measured by spectrophotometer NanoDrop One (Thermo Fisher).

#### Plasmid constructs

Full-length or partial coding sequence of zebraifish *dopey2* (XM_017357831.2) and *pcdh7b* (XM_678029.8) was amplified by Q5 High-Fidelity DNA Polymerase (NEB) and cloned into vectors. Primers are listed in Table S1. These PCR products were verified by agarose gel electrophoresis. PCR purification or DNA gel extraction (TIANGEN) was performed to obtain clean PCR products. Correct PCR products were cloned into pcDNA3.1(+) vector or pGEM-T Easy vector (Promega) by T4 DNA ligase (NEB). Recombinant plasmids were introduced into *E. coli* TOP10 chemically competent cells and sager sequencing to validate them. Successfully recombined plasmids were amplified and purified from bacterial overnight cultured, then purified by silica-based nucleic acid isolation kits (MACHEREY-NAGEL).

#### Morpholino and mRNA injection

Morpholino (MO) antisense oligonucleotides were purchased from Gene Tools LLC (Oregon, USA). Dopey2 MO was designed to be complementary to the sequence near the translation start site, the sequence is: 5′-CATTCTACCTGGGCCTCCTCGTCCT-3'. The MO design of Pcdh7b is complementary to the sequence near the splicing site of the first exon and intron, and the sequence is: 5′-AGCAGAAATGTTGTTCTTACCTGTT-3′. Pcdh7b MO-AUG was designed to be complementary to the sequence near the translation start site, the sequence is: 5′-CCACAATGCCTGTAGTCCTCATAGC-3'. Pcdh7a MO was designed to be complementary to the sequence near the translation start site, the sequence is: 5′-TGGTGTTGTGTCTCACCTGTGCATC-3′, The standard control MO sequence is 5'- TGCTGGAAAAGCCGCCAGGAGCTAT-3′. Capped mRNA was first cloned into pcDNA3.1(+) vector, linearized, synthesized by mMESSAGE mMACHINE T7 Transcription Kit (Ambion). The volume of morpholino solution injected into zebrafish embryos was 1 nL, the concentration of morpholino solution was 1–5 ng/nL, and the concentration of mRNA was 50–150 ng/μL.

#### Whole-mount*in situ* hybridization

Whole-mount*in situ* hybridization (WISH) for zebrafish embryos was performed following standard protocol (by Thisse’s). Using the linearized plasmid as a template, an RNA labeling kit (Roche) was used to synthesize digoxigenin-UTP-labeled antisense RNA probes *in vitro*. Embryos were permeabilized with proteinase K (Promega) and hybridized overnight at 65°C. After *in situ* hybridization, the embryos were immersed in glycerol and photographed with a stereomicroscope.

#### Zebrafish whole-mount immunofluorescence staining and frozen section immunofluorescence staining

Zebrafish embryos were harvested and fixed in 4% paraformaldehyde overnight, washed twice with PBS and once with ultrapure water for 5 minutes each, placed in acetone at −20°C for 7 minutes, then washed with ultrapure water once and PBS Twice for 5 minutes each, placed in PBST. Embryos were blocked with 3% BSA in PBST for 1 hour. Next, embryos were treated with primary antibody p-histone H3 (S10) antibody (Millipore, 04-817, 1:500 dilution) at 4°C overnight. After three washes with PBST, fluorescent secondary antibodies (Thermo Fisher, 1:800 dilution) were incubated for 2 hours. DAPI (Invitrogen, 1:3000 dilution) was incubated for 5 min. Stained embryos were placed in 2% methylcellulose and photographed by fluorescence microscope.

After the zebrafish embryos were fixed, they were embedded in agarose blocks and dehydrated with sucrose. The blocks were sliced with freezing microtome at a thickness of 5 μm and place on glass slides. Immunofluorescence staining was performed following standard procedures. The following primary antibodies were used: Sox2 (Abcam, ab97959, 1:500 dilution), p-histone H3 (S10) antibody (Millipore, 04-817, 1:500 dilution), Dopey2 (HUABIO, 0903-3, 1:500 dilution, anti-mouse monoclonal antibody and anti-human polyclonal antibody were used), BrdU (Sigma, ab6326, 1:500 dilution). The cells were then incubated with fluorescent secondary antibody (Thermo Fisher, 1:800 dilution) incubated for 2 hours at room temperature. DAPI (Thermo Fisher, 1:3000 dilution) was incubated for 5 min. Photographs were taken with a fluorescence microscope after mounting.

#### 5-bromo-20-deoxyuridine (BrdU) Pulse Labeling

Zebrafish embryos were incubated with 0.1 mg/mL BrdU (Sigma) for 2 hours in an incubator when they developed to 22 hpf (hour post-fertilization). When the embryos developed to 24 hpf, the embryos were collected into 1.5 mL EP tubes, 30 embryos per tube, and fixed with 4% paraformaldehyde in a 4°C refrigerator overnight. Prior to immunostaining, zebrafish embryo sections were incubated with HCl (1 N) on ice for 10 minutes to rupture the DNA structure of the labeled cells, then treated with HCl (2 N) for 10 minutes at room temperature and 20 minutes at 37°C. After the acid washes, these embryos were added borate buffer (0.1 M) and incubated at room temperature for 12 min. Zebrafish embryos were then rinsed in PBS/glycine (1 M). Subsequentstep is the same as zebrafish whole-mount immunofluorescence staining.

#### Western blotting analysis

24 hpf embryos were dechorionated and homogenized in RIPA buffer containing protease inhibitor cocktail (Thermo Fisher) and prepared for SDS-PAGE. Signal detection with ɑ-tublin (Abcam, ab15246, 1:1000 dilution), Pcdh7 (Abcam, ab139274, 1:500 dilution), Dopey2 (Huabio, 0903-3, 1:500 dilution, anti-mouse monoclonal antibody and anti-human polyclonal antibody were used) overnight at 4°C, followed by incubation with appropriate secondary antibodies (Zsbio, 1:5000 dilution). Immunoblotting was performed according to standard procedures.

#### Co-immunoprecipitation

To investigate whether Dopey2 interacts with Pcdh7 and the changes in ubiquitination of Pcdh7, HEK293T cells or zebrafish embryos were homogenized in IP buffer (50 mM Tris-HCl pH 7.4, 150 mM NaCl, 2 mM EDTA, 1% NP-40) containing protease inhibitor cocktail (Thermo Fisher). After centrifugation at 4°C, the supernatant was collected and combined with beads (Invitrogen) and antibody Pcdh7 (Santa Cruz, sc-517042, 1:50 dilution) overnight. After rinsing with wash buffer, subsequent analysis was performed according to the Western blotting method described above. Band intensities were measured with ImageJ software.

#### Quantitative RT-PCR

Zebrafish embryos were harvested at the desired period and dechorionated. Total RNA was extracted with TRIZOL and treated with TURBO DNase (Invitrogen), and then used to synthesized cDNA with a reverse transcription kit (TAKARA). SYBR green (ROCHE) kit were used for qQPCR. Each sample was tested in triplicate and BioRad CFX Manager software was used for data analysis. The cycle threshold (Ct) value was obtained. The difference between the Ct values of the target gene and the housekeeping gene was expressed as ΔCt value. The ΔΔCt value was obtained by subtracting the ΔCt value of control sample from ΔCt value of the experimental sample. Relativefold changes in gene expression were calculated as 2^−ΔΔCt^. The sequences of qRT-PCR primers are listed in TableS1.

#### Chromosome immunoprecipitation assay

200 Morpholino-injected embryos were collected at 48 hpf, and fixed in 1% formaldehyde, then homogenized and sonicated into 300-500 bp chromatin fragments. Chromatin fragments were immunoprecipitated overnight with Anti-RNA polymerase II CTD repeat YSPTSP and S5 antibody (Abcam, ab817 and ab5408, 1 ug per 0.5 ml of sonicated chromatin) or IgG (Santa Cruz, 1ug per 0.5 ml of sonicated chromatin). Chromatin Immunoprecipitation (ChIP) Assay Kit (Beyotime) was used to obtain targeted chromatin fragments. Nucleic acids were purified by DNA purification kit (TIANGEN) and analyzed by Quantitative RT-PCR according to the previous procedure. The sequences of qRT-PCR primers are listed in TableS1.

### Quantification and statistical analysis

All quantitative experiments were performed with at least three independent biological repeats. Results were expressed as means ± SEM. Significance of differences between means was evaluated by Student’s *t* test. ∗∗∗p < 0.001, ∗∗p < 0.01, ∗p < 0.05; NS, not significant.

## Data Availability

• Raw and analyzed data reported in this paper will be shared by the lead contact upon request. • This paper does not report original code. • Any additional information required to reanalyze the data reported in this paper is available from the lead contact upon request.
