# Supplementary material for: Dopey2 and Pcdh7 orchestrate the development of embryonic neural stem cells/ progenitors in zebrafish
Source: iScience. 2023 Feb 25;26(3):106273. doi: 10.1016/j.isci.2023.106273 (PMC10014312; doi:10.1016/j.isci.2023.106273)
Supplement: Document S1. Figures S1–S8 and Table S1 [file mmc1.pdf]

**iScience, Volume *26***

## Supplemental information

# Dopey2 and Pcdh7 orchestrate the development of embryonic neural stem cells/ progenitors in zebrafish

**Yue Xiao, Min Hu, Qiyan Lin, Ting Zhang, Siying Li, Linjuan Shu, Xiuli Song, Xiaoyong Xu, Wentong Meng, Xue Li, Hong Xu, and Xianming Mo**

**Dopey2 and Pcdh7 orchestrate the development of embryonic neural stem cells/ progenitors in zebrafish**

Yue Xiao[1], Min Hu[1], Qiyan Lin[1], Ting Zhang[1], Siying Li[1], Linjuan Shu[1], Xiuli Song[2], Xiaoyong Xu[2], Wentong Meng[1], Xue Li[1], Hong Xu[1], Xianming Mo[1,*]
[1]Department of Pediatric Surgery, Laboratory of Stem Cell Biology, State Key Laboratory of Biotherapy, West China Hospital, West China Medical School, Sichuan University, Chengdu 610041, China.
[2]Hangzhou HuaAn Biotechnology Co.Ltd, Hangzhou, China
[*]Correspondence: xmingmo@scu.edu.cn

Table S1. Sequences of primers used in this study, related to STAR Methods

Sequences of primers for quantitative RT-PCR analysis of gene expression

| | Sequence (in 5' to 3' direction) | |
|---|---|---|
| Gene names | sense-primer | antisense-primer |
| *sox2* | CGAGTCTAGTTCGAGTCCGC | GTTAATCGTCGTACCGGGCA |
| *nestin* | CAGGAGGCAGCCAACAACTA | GGGTGTTTACTTGGGCCTGA |
| *huc* | GCCAGCTACGGAGTCAAGAG | CATGGTGACGAAGCCAAAGC |
| *dopey2* | ACCAGTCGTAGCACCTCTCT | CCTCCCCCTCTGTCTCATCA |
| *olig2* | TTCCATCCGTCCAGTTGTGG | ACATGCTACACGGACAAGGG |
| *pcdh7b* | GCCGATATCGCTCGGTCAAC | GGGGCCGAGAGTCTGTTTGT |

Sequences of primers for WISH probs of gene expression

| | Sequence (in 5' to 3' direction) | |
|---|---|---|
| Gene names | sense-primer | antisense-primer |
| *sox2* | ACAATTCGGAAATAAGTAAGCG | GTAGGTCTGCGAGTTGGTCAT |
| *nestin* | TTGGGCCGTGTGAAACTTCT | AGGCTTGAGCTGCCTTGTAG |
| *huc* | AGCAGAGGCACAGAAGGAGC | TGAGGCAGGTAGTTGACGAT |
| *olig2* | CACTGAACGCCATGGACTCT | ACATGCTACACGGACAAGGG |
| *dopey2* | TGCAGTGGACAGATGCTGAG | AGCGCCCCATAAAACACAGA |
| *pcdh7b* | AACGAGCGGCGAATAGACCG | TCAGAATGGCTTGGGAGGAG |

Sequences of primers for PCR cloning

Sequence (in 5' to 3' direction)

| Gene names | sense-primer | antisense-primer |
|---|---|---|
| *dopey2* | CTGAGCATGCTGGGACATGA | GCAAAGCCCACATTAAGCCC |
| *dopey2* TRUN-C | CTGAGCATGCTGGGACATGA | GAGCTTGAGCGGTGAGACTT |
| *dopey2* TRUN-N | CCATCTGCATCAATCTGTGAGG | GCAAAGCCCACATTAAGCCC |
| *pcdh7b* | GTCAAGTCATGGCAAACACG | GCAAGGCTTCAGCAAAGTAG |
| *pcdh7b* TRUN-C | GGATTGAATATCTGGCTTCG | ACATCCTGCATGAACTTTGG |
| *pcdh7b* TRUN-N | GAGTATGTTTTCGGAGCAGCCA | GCAAGGCTTCAGCAAAGTAG |

Sequences of primers for quantitative RT-PCR analysis for chromosome immunoprecipitation assay

| | Sequence (in 5' to 3' direction) | |
|---|---|---|
| Primer names | sense-primer | antisense-primer |
| Dopey2 Pirmer P1 | GTCGACTTTACACCCCTCGT | AAAGCGGAGAGCAGCTAGAA |
| Dopey2 Primer P2 | TTGTCGACTTTACACCCCTCG | AAGCGGAGAGCAGCTAGAAT |
| Dopey2 Primer P3 | CAGTAACTCTATCCTGCCTTGA | AGCATGCTCAGCATCTGTCT |
| Dopey2 Primer P4 | GACATGACTGAGGCGTGATG | GAGATCGGCCCATTCACTAG |
| GAPDH Primer P1 | AAAAGACCCCACCCGATGC | AGCTTGTTCGGTGCAATCATTA |
| GAPDH Primer P2 | AATGATTGCACCGAACAAGC | TGTCTGTTAACAACTTGCGATGG |

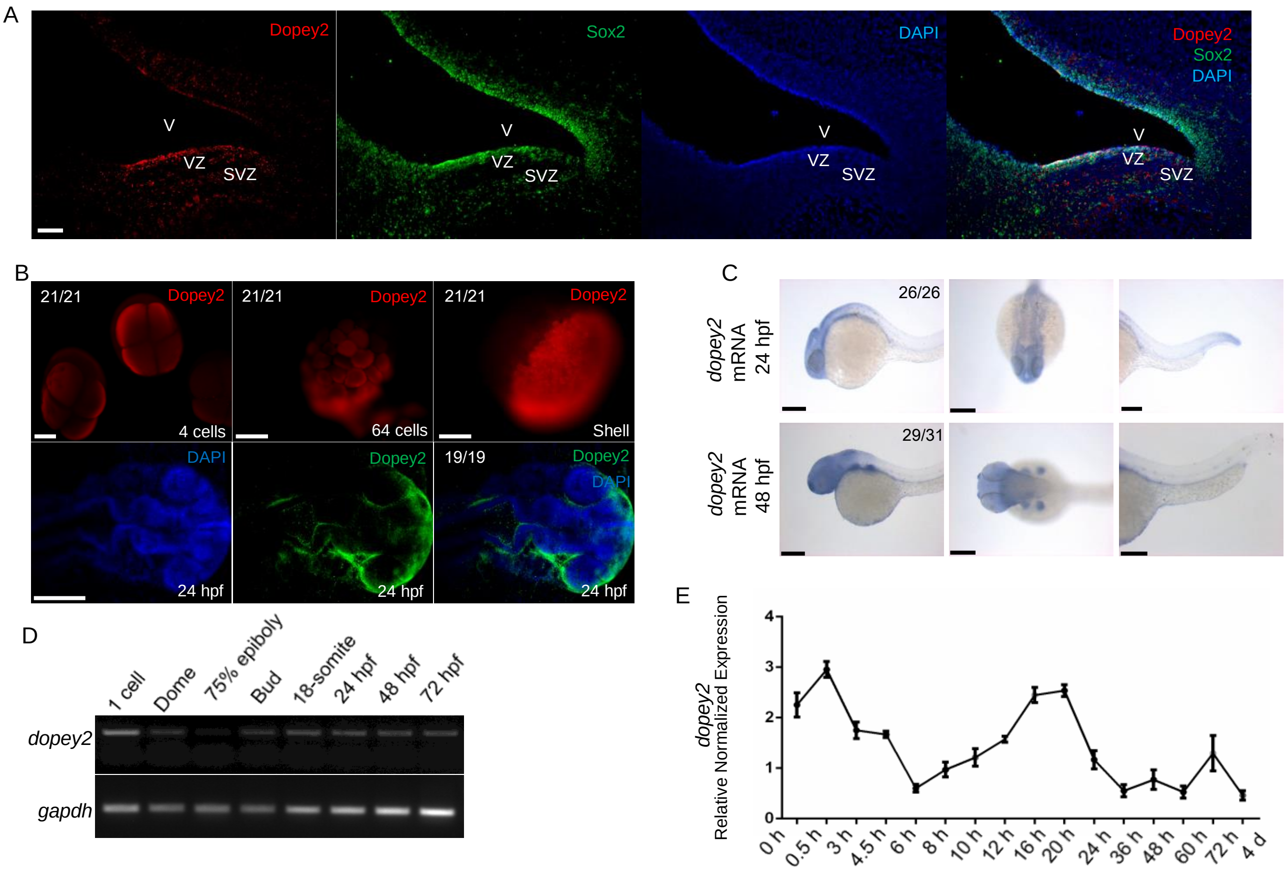


**Figure S1. Dopey2 expressed in the brains of mice and zebrafish embryos, related to Figure 1.**

(A) Representative images of E15 mouse brain sections stained for Dopey2 (red), Sox2 (green), DAPI (blue). V, ventricle; VZ, ventricular zone; SVZ, subventricular zone. Scale bar = 100 μm.

(B) Whole-mount immunofluorescence staining detected Dopey2 in zebrafish embryos at different development stages. Scale bar = 200 μm.

(C) *In situ* hybridization analysis detected *dopey2* expressing pattern in zebrafish embryos brain and tail by dorsal or lateral sight at 24 hpf and 48 hpf. Scale bar = 200 μm.

(D) Semi-Quantitative RT-PCR analysis detected *dopey2* expression at different developmental stages. Semi-Quantitative RT-PCR analysis of *gapdh* expression was used as the loading control.

(E) Real-time fluorescence quantitative PCR analysis revealed the expression quantity of *dopey2* gene at different developmental stages from 0 hpf to 4 dpf.

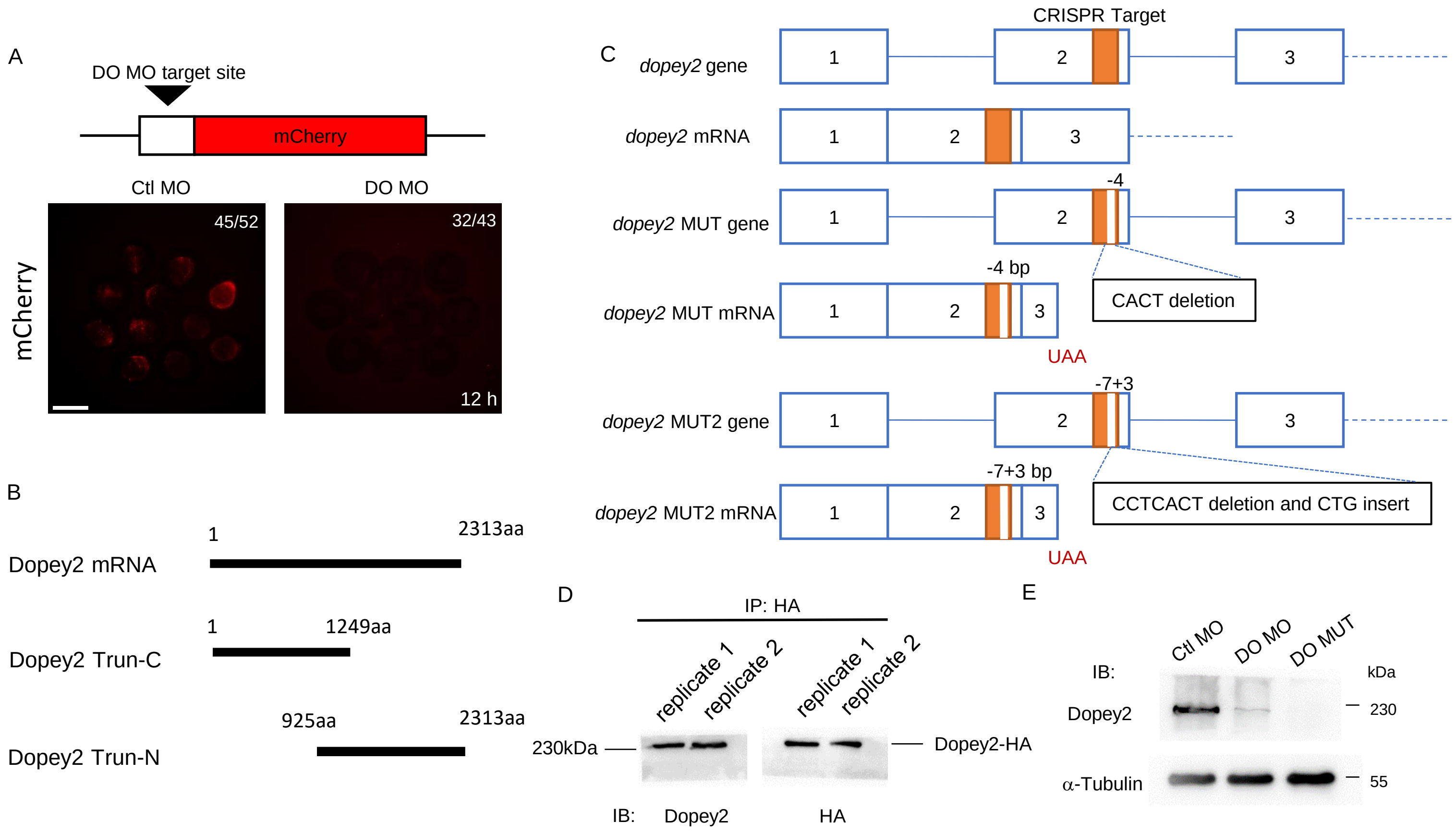


**Figure S2. Efficient inactivation of Dopey2 function using different methods, related to Figure 2.**

(A) DO MO target site was fused with mCherry to create a recombinant construct. Upon injecting the recombinant plasmid along with Ctl MO and DO MO into embryos, DO MO was able to suppress the expression of fluorescence. Scale bar = 500 μm.

(B) The illustration of Dopey2 truncated protein, DO TRUN-C refers to the shortened form of Dopey2 protein that results from cutting off the C-terminal end. TRUN-N represents the truncated version of Dopey2 protein at its N-terminal end.

(C) The schematic presentation of CRISPR/Cas9-mediated genome editing for the dopey2 gene and making premature mRNA mutants.

(D) Anti-human Dopey2 antibody was able to detect the zebrafish Dopey2-HA fused protein.

(E) Immunoblotting detected Dopey2 protein levels in 48 hpf WT embryos pre-injected with Ctl MO or DO MO and DO MUT embryos. Immunoblotting of α-tubulin was used as loading control.

Ctl MO: control MO, DO MO: *dopey2* MO, DO TRUN: *dopey2* truncated RNA.

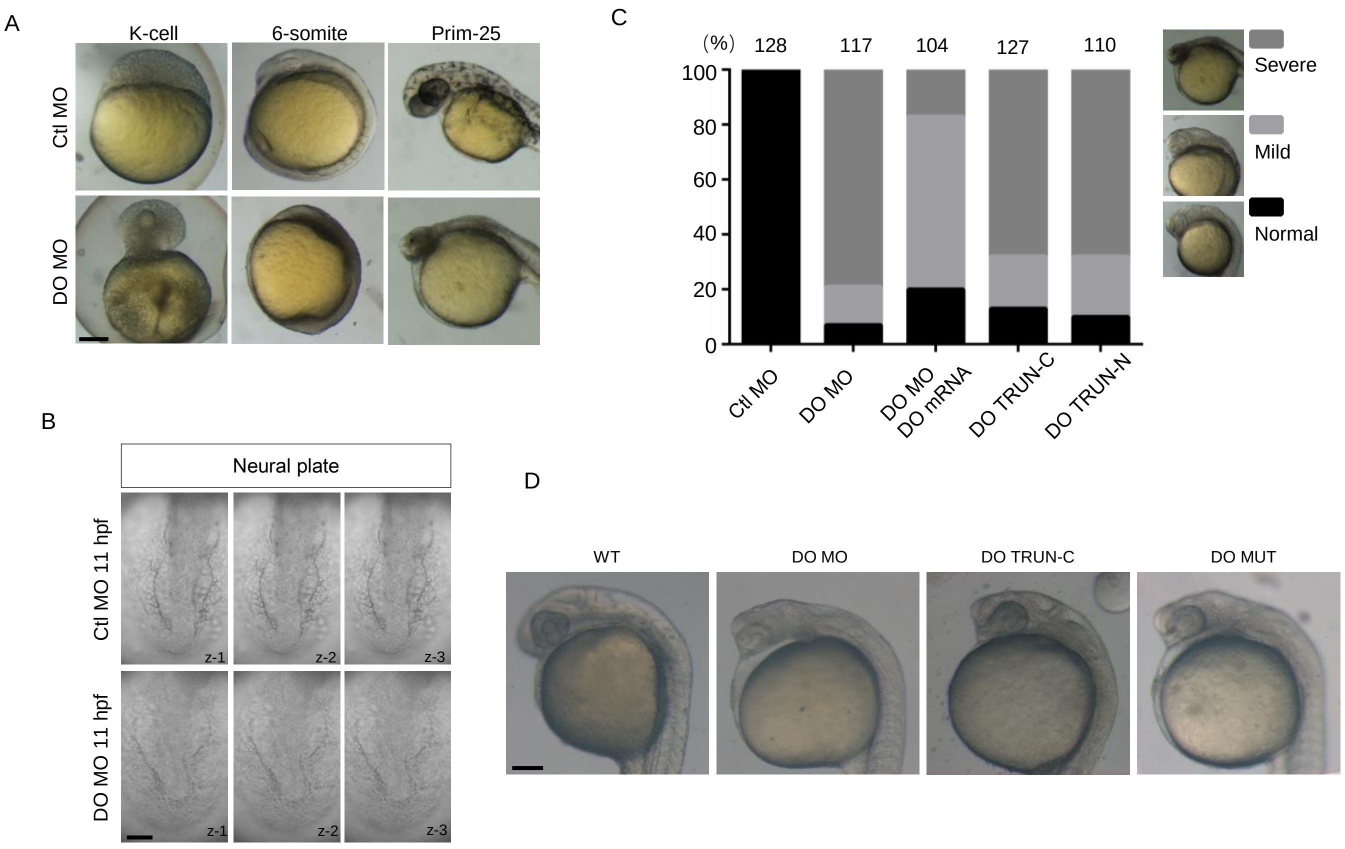


**Figure S3. Knocking down Dopey2 expression induced defects in zebrafish embryos, related to Figure 2.**

(A) Development process of zebrafish embryos pre-injected with Ctl MO or DO MO from K-cell to Prim-25 stage. Scale bar = 200 μm.

(B) Early morphogenesis of neural plate in embryos pre-injected with Ctl MO or DO MO. Scale bar = 100 μm.

(C) Left panel indicates ratio of phenotypes in each group injected with Ctl MO (4 ng/nl, n = 128), DO MO (4 ng/nl, n = 117), DO MO+DO mRNA (DO MO 4 ng/nl + DO mRNA 50 ng/nl, n = 104), DO TRUN-C (DO TRUN-C mRNA 150 ng/nl, n = 127) , DO TRUN-N (DO TRUN-NS mRNA 150 ng/nl, n = 110). Right panel shows different phenotype of MO pre-injected embryos at 24 hpf.

(D) Phenotypes of WT, WT injected DO MO or DO TRUN-C and DO MUT zebrafish embryos at 24 hpf. Scale bar = 200 μm.

Ctl MO: control MO, DO MO: *dopey2* MO, DO TRUN: *dopey2* truncated RNA, DO mRNA: *dopey2* mRNA.

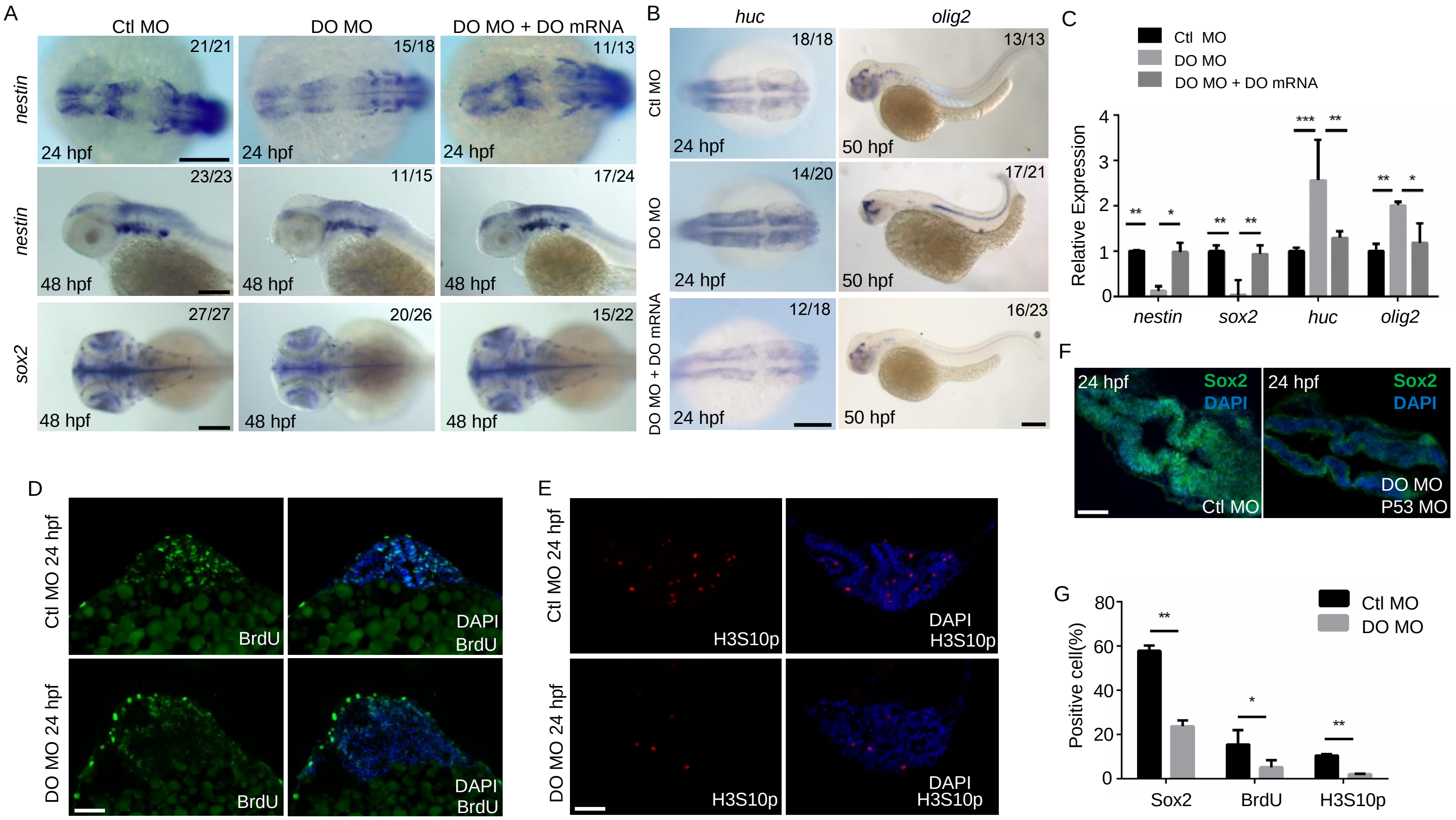


**Figure S4. Dopey2 modulates the proliferation of neural stem cells/progenitors and inhibits the differentiation of neural stem cells/progenitors in zebrafish embryonic brains, related to Figure 2.**

(A, B) In situ hybridization analysis showed the expression of nestin, sox2, huc and olig2 genes. Embryos were analyzed at 24 hpf (hour post-fertilization), 48 hpf and 50 hpf, pre-injected with Ctl MO, DO MO and DO MO + DO RNA. Scale bar = 200 μm.

(C) Real-time fluorescence quantitative PCR analysis showed the relative expression level of nestin, sox2, huc and olig2 genes in embryos at 48 hpf, pre-inject with Ctl MO, DO MO and DO MO + DO mRNA (mean ± s.e.m, n = 3, Student's t test: ***P < 0.001, **P < 0.01, *P < 0.05).

(D, E) Representative immunofluorescence staining of BrdU and H3S10p in the frozen sections of 24 hpf embryos pre-injected with Ctl MO or DO MO. Scale bar = 50 μm.

(F) Representative immunofluorescence staining for Sox2 in frozen sections of 24 hpf embryos pre-inject with Ctl MO or DO MO. Scale bar = 50 μm.

(G) Diagram showed quantitative analysis of Sox2, BrdU and H3S10p positive cells in embryo sections pre-injected with Ctl MO or DO MO (mean ± s.e.m, n = 3, Student's t test: **P < 0.01, *P < 0.05). Images were quantified by ImageJ software.

Ctl MO: control MO, DO MO: *dopey2* MO, DO mRNA: *dopey2* mRNA

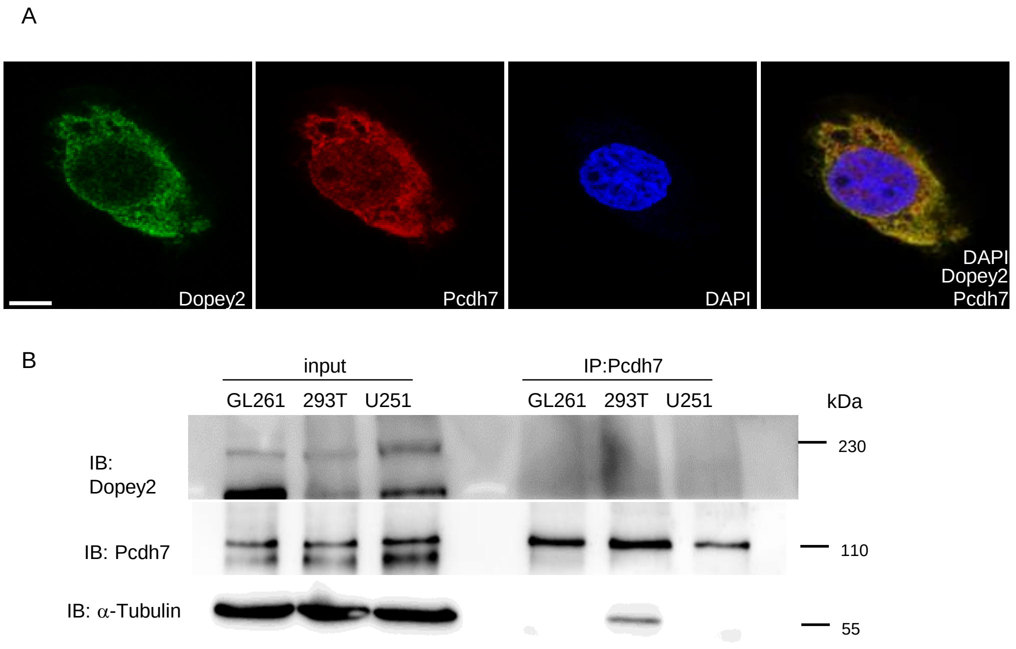


**Figure S5. Dopey2 did not combine with Pcdh7 in cells, related to Figure 3.**

(A) Immnofluorescence for Dopey2 and Pcdh7 in U251 cells. Results showed they don't localize together in cells. Scale bar = 10 μm.

(B) Coimmunoprecipitation (co-IP) of Pcdh7 with Dopey2 in GL261, 293T and U251 cells. Western blot analysis of immunoprecipitated proteins and input lysate showed Dopey2 don't interact with Pcdh7.

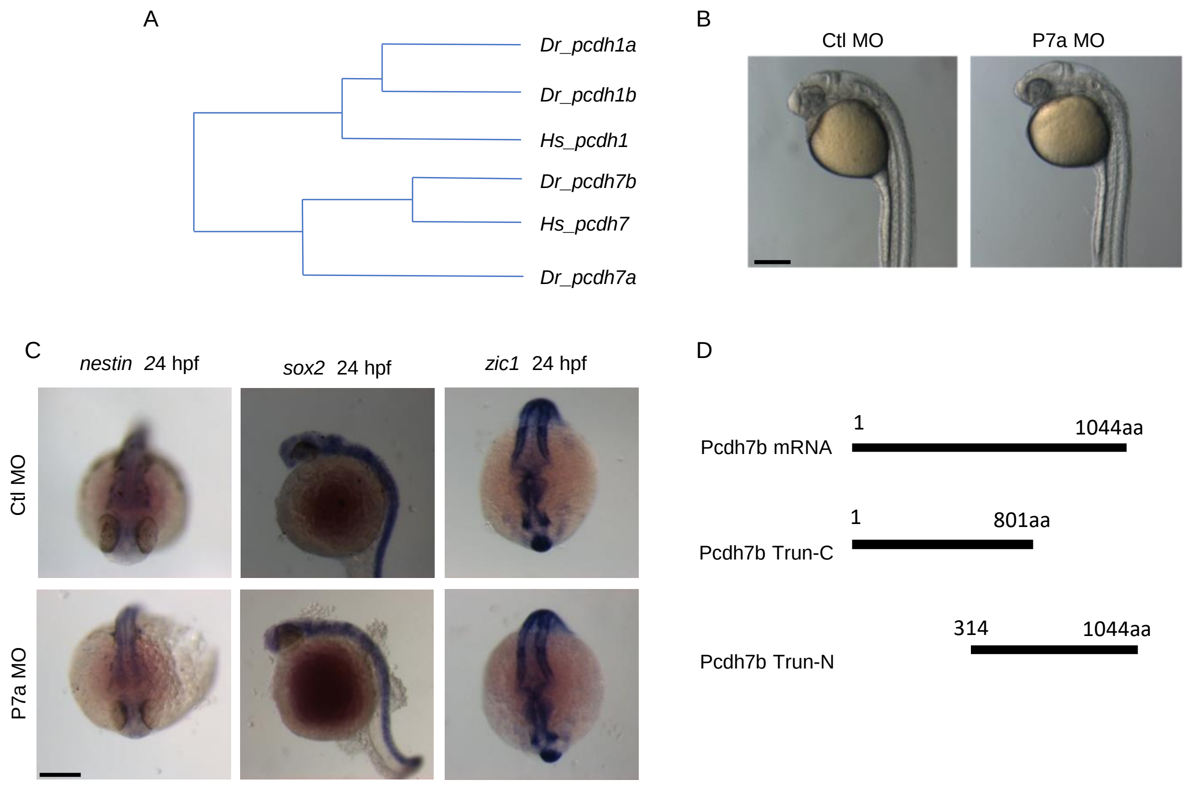


**Figure S6. Different methods were employed to disrupt the function of Pcdh7b, related to Figure 4.**

(A) Phylogenetic relationships between the zebrafish and human *pcdh1*, *pcdh7* genes.

(B) Phenotypes of Ctl MO or P7a MO injected zebrafish embryos at 26 hpf. Scale bar = 200 µm.

(C) In situ hybridization analysis showed the expression of *nestin*, *sox2*, *zic1* genes. Embryos were analyzed at 24 hpf, pre-injected with Ctl MO or P7a MO. Scale bar = 200 µm.

(D) The illustration of Pcdh7 truncated protein, P7 TRUN-C refers to the shortened form of Pcdh7 protein that results from cutting off the C-terminal end. TRUN-N represents the truncated version of Pcdh7 protein at its N-terminal end.

Ctl MO: control MO, P7a MO: *pcdh7a* MO, P7 TRUN: *pcdh7b* truncated RNA.

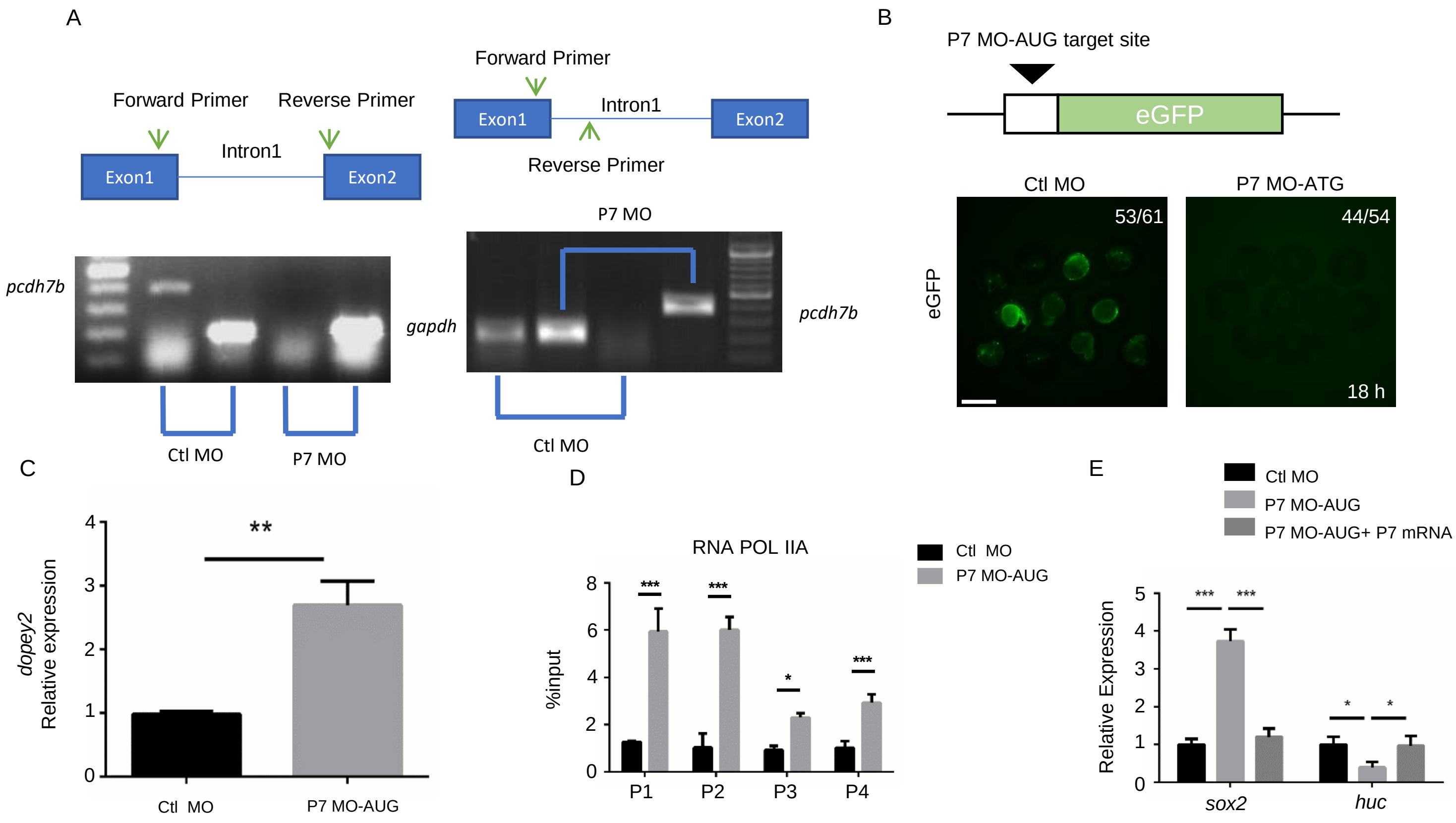


**Figure S7. P7 MO effectively knocks down P7 expression in zebrafish, related to Figure 6.**
(A) Schematic presentation of primers designed for detecting efficiency of RNA splicing by blocked by Pcdh7b MO. Left panel: Semi-Quantitative RT-PCR showed P7 MO injected embryos did not form the specific band by primers in exon1 and exon2. Right semi-Quantitative RT-PCR showed Ctl MO injected embryos couldn't form the specific band by primers in exon1 and intron1 but P7 MO injected embryos could. Because P7 MO is complementary to the sequence near the splicing site of the first exon and intron, these results represented P7 MO did specifically block pcdh7b pre-mRNA splicing.
(B) P7 MO-AUG target site was fused with eGFP to create a recombinant construct. Upon injecting the recombinant plasmid along with Ctl MO and P7 MO-AUG into embryos, P7 MO-AUG was able to suppress the expression of fluorescence. Scale bar = 500 μm.
(C) Real-time fluorescence quantitative PCR analysis detected the relative expression level of *dopey2* gene in 48 hpf embryos pre-injected with Ctl MO or P7 MO-AUG (mean $\pm$ s.e.m, n = 3, Student's t test: **P < 0.01).
(D) ChIP analysis results of 48 hpf embryos pre-injected with Ctl MO or P7 MO-AUG (mean $\pm$ s.e.m, n = 3, Student's t test: ***P < 0.001, *P < 0.05).
(E) Real-time fluorescence quantitative PCR analysis detected the relative expression level of *sox2* and *huc* gene in 48 hpf embryos pre-injected with Ctl MO, P7 MO-AUG or P7 MO-AUG + P7 mRNA (mean $\pm$ s.e.m, n = 3, Student's t test: ***P < 0.001, *P < 0.05).
WT: wild type, Ctl MO: control MO, P7 MO: *pcdh7b* MO, P7 MO-AUG: *pcdh7b* MO-AUG, RNA POL IIA: RNA polymerase II with an unphosphorylated CTD.

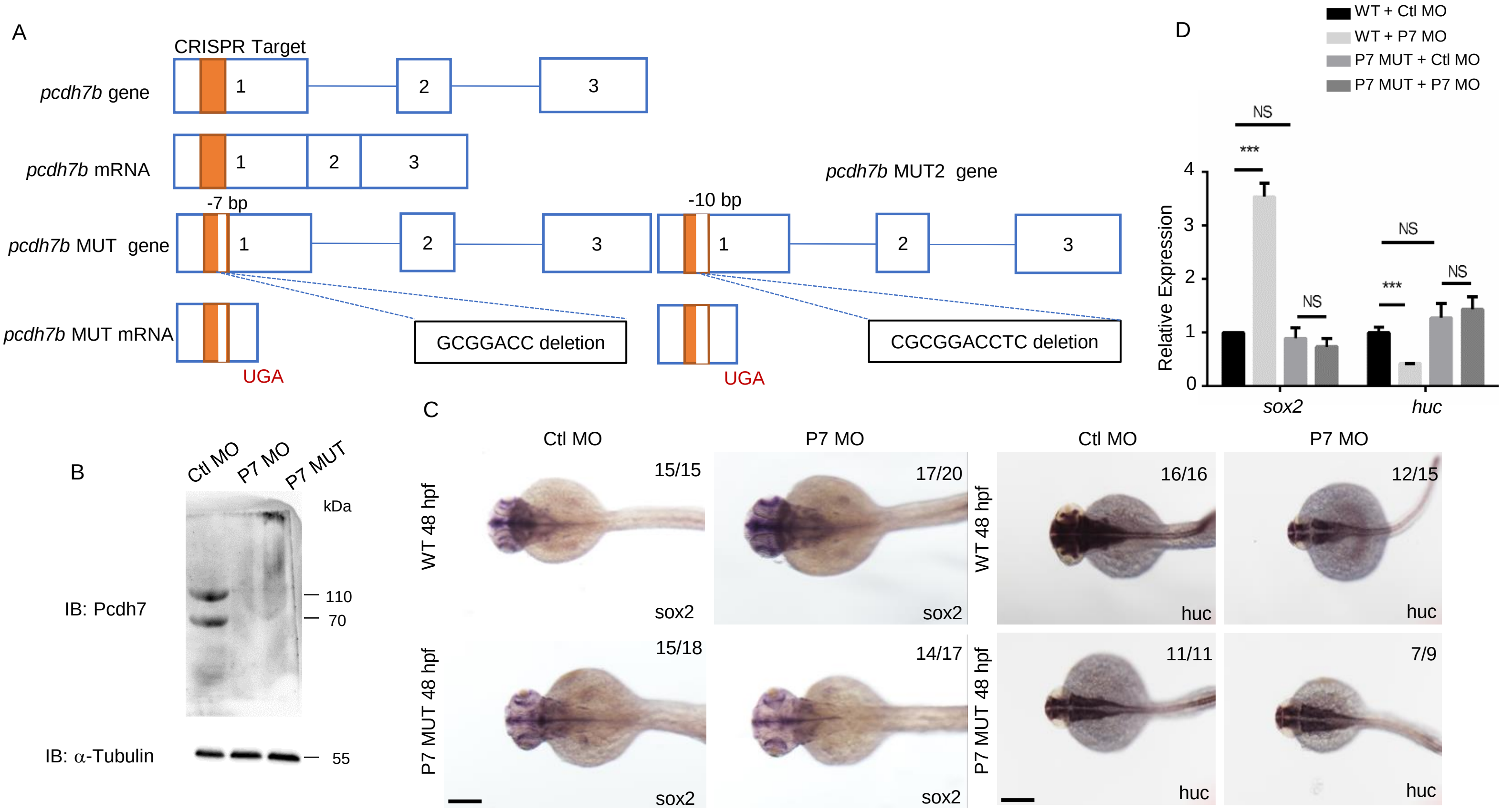


**Figure S8. The P7 MUT did not show any visible characteristics as a result of genetic compensation mechanisms, related to Figure 6.**

(A) A schematic presentation of CRISPR/Cas9-mediated genome editing for the *pcdh7b* gene and produced premature mRNA mutants.

(B) Immunoblotting detected Pcdh7 protein levels in 48 hpf WT embryos pre-injected with Ctl MO or P7 MO and P7 MUT embryos. Immunoblotting of α-tubulin was used as loading control.

(C)*In situ* hybridization analysis showed the expression of *sox2*, *huc* genes. Embryos were analyzed at 48 hpf (hour post-fertilization). WT or P7 MO embryos were pre-injected with Ctl MO or p7 MO. Scale bar = 200 μm.

(D) Real-time fluorescence quantitative PCR analysis showed the relative expression level of *sox2*, *huc* genes in embryos at 48 hpf, WT or P7 MUT embryos were pre-injected with Ctl MO or P7 MO (mean ± s.e.m, n = 3, Student's t test: ***$P < 0.001$, NS = not significant).

Ctl MO: control MO, P7 MO: *pcdh7b* MO, WT: wild type, P7 MUT: *pcdh7b* mutant.

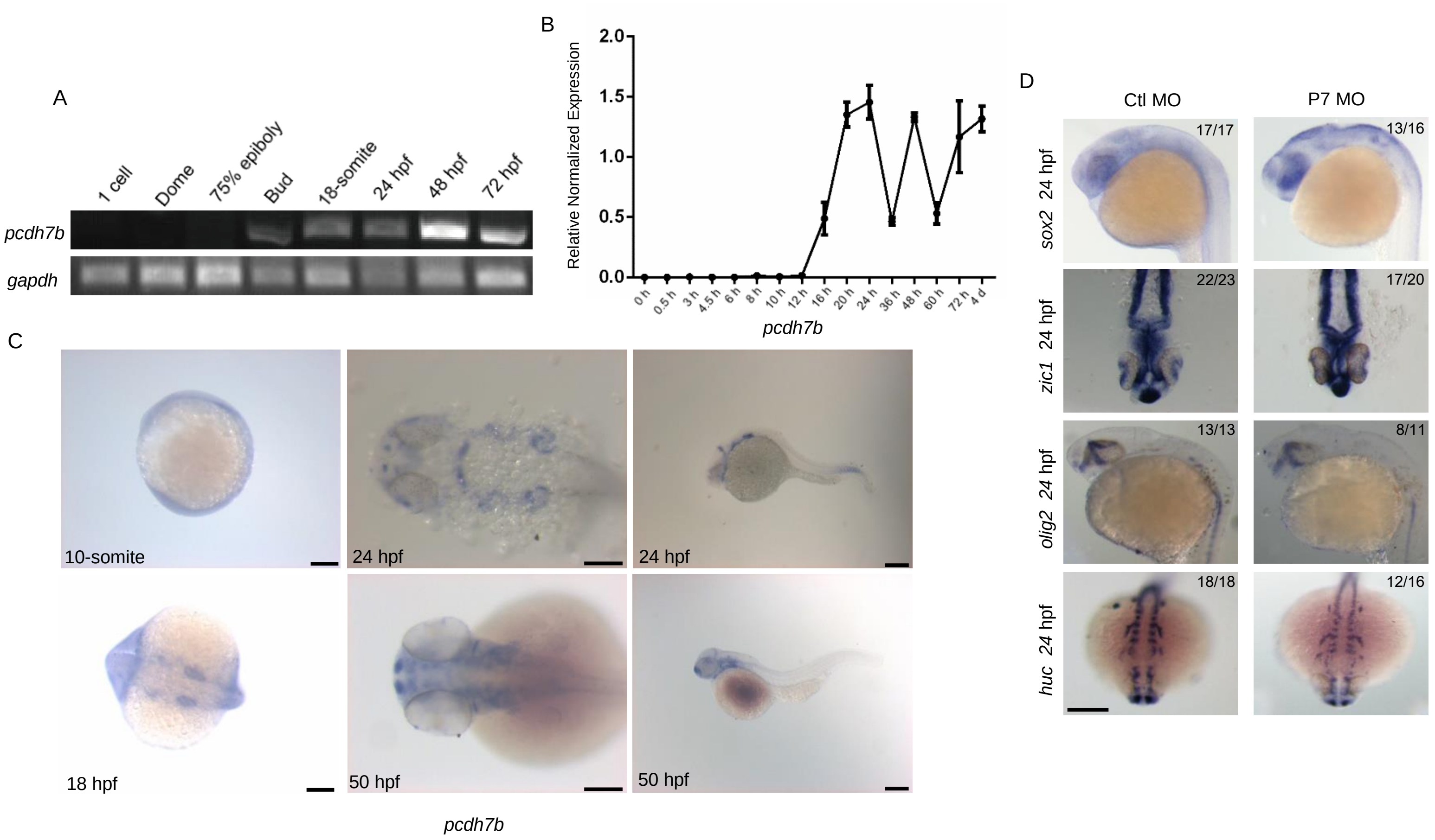


**Figure S9. The temporal and spatial expression pattern of *pcdh7b* gene in zebrafish embryos, related to Figure 6.**

(A) Semi-Quantitative RT-PCR analysis detected *pcdh7b* expression at different developmental stages of zebrafish embryos. Semi-Quantitative RT-PCR analysis of *gapdh* expression was used as the loading control.

(B) Real-time fluorescence quantitative PCR analysis revealed *pcdh7b* expression quantity at different developmental stages of zebrafish embryos from 0 hpf to 4 dpf.

(C) *In situ* hybridization analysis detected the expression of *pcdh7b* in zebrafish embryos at different development stage. Scale bar = 200 μm.

(D) *In situ* hybridization analysis detected *nestin* gene in embryos pre-injected with Ctl MO, P7 MO or P7 TRUN-C. Scale bar = 200 μm.

Ctl MO: control MO, P7 MO: *pcdh7b* MO, P7 TRUN: *pcdh7b* truncated RNA.
